# Factors associated with mammography use: A side‐by‐side comparison of results from two national surveys

**DOI:** 10.1002/cam4.3128

**Published:** 2020-07-17

**Authors:** Lihua Li, Jiayi Ji, Melanie Besculides, Nina Bickell, Laurie R. Margolies, Lina Jandorf, Emanuela Taioli, Madhu Mazumdar, Bian Liu

**Affiliations:** ^1^ Department of Population Health Science and Policy Icahn School of Medicine at Mount Sinai New York NY USA; ^2^ Institute for Healthcare Delivery Science Mount Sinai Health System New York NY USA; ^3^ Tisch Cancer Institute Icahn School of Medicine at Mount Sinai New York NY USA; ^4^ Department of Diagnostic, Molecular and Interventional Radiology Icahn School of Medicine at Mount Sinai New York NY USA; ^5^ Institute for Translational Epidemiology Icahn School of Medicine at Mount Sinai New York NY USA

**Keywords:** behavioral risk factor surveillance system, breast cancer screening, mammography use, National health interview survey, predictive margins, random forest

## Abstract

**Background:**

Mammography use is affected by multiple factors that may change as public health interventions are implemented. We examined two nationally representative, population‐based surveys to seek consensus and identify inconsistencies in factors associated with mammography use in the entirety of the US population, and by black and white subgroups.

**Methods:**

Self‐reported mammography use in the past year was extracted for 12 639 and 169 116 women aged 40‐74 years from the 2016 National Health Interview Survey (NHIS) and the 2016 Behavioral Risk Factor Surveillance System (BRFSS), respectively. We applied a random forest algorithm to identify the risk factors of mammography use and used a subset of them in multivariable survey logistic regressions to examine their associations with mammography use, reporting predictive margins and effect sizes.

**Results:**

The weighted prevalence of past year mammography use was comparable across surveys: 54.31% overall, 54.50% in white, and 61.57% in black in NHIS and 53.24% overall, 56.97% in white, and 62.11% in black in BRFSS. Overall, mammography use was positively associated with black race, older age, higher income, and having health insurance, while negatively associated with having three or more children at home and residing in the Western region of the US. Overweight and moderate obesity were significantly associated with increased mammography use among black women (NHIS), while severe obesity was significantly associated with decreased mammography use among white women (BRFSS).

**Conclusion:**

We found higher mammography use among black women than white women, a change in the historical trend. We also identified high parity as a risk factor for mammography use, which suggests a potential subpopulation to target with interventions aimed at increasing mammography use.

## Graphical abstract

Different from the historical trend, we found higher mammography use among black women than white women. We identified high parity as a risk factor for mammography use, which is less known and suggests a potential subpopulation for targeted interventions.

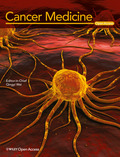

## 1 INTRODUCTION

Screening mammography has been shown to decrease breast cancer mortality.^1^ As breast cancer is the most common cancer in women, ^2^ screening mammography use has been a long‐standing national health‐care objective.^3^ Mammography use, therefore, has been incorporated in many large‐scale national surveys, including the National Health Interview Survey (NHIS) and the Behavioral Risk Factor Surveillance System (BRFSS). Both surveys provide nationally representative samples for examining the prevalence and temporal trends of mammography use, identifying risk factors associated with mammography use, and investigating disparities in use among population subgroups.^4^, ^5^, ^6^, ^7^ Four common domains of factors shared by NHIS and BRFSS are: sociodemographics, health status, health‐care access and utilization, and health behaviors.^6^

Overlap of available variables from both surveys provides a great opportunity to conduct a side‐by‐side comparison between NHIS and BRFSS data. Such a study can help strengthen our understanding of the extent of the associations between risk factors and mammography use and results from one survey can be verified by the other. Additionally, such a comparison study can help clarify inconsistent results in the literature regarding the effects of certain risk factors on mammography use. For example, while some studies have found severely obese women, regardless of black or white race, to be significantly less likely to undergo mammography, others have found no association between mammography use and body mass index (BMI) in black women.^8^, ^9^, ^10^

Understandably, discrepancies among studies based on different national surveys are to be expected, given variations in the survey design, survey mode, the construct of survey questions, and weighting methods.^11^, ^12^ In addition, researchers may select different sets of risk factors when studying their impact on mammography use. Moreover, specific risk factors can change as recommendations and public health interventions are updated and implemented. For example, the biennial screening mammography recommendation by the United States Preventive Services Task Force (USPSTF) started in 2009. In the 2016 and 2009 guidelines, mammography screening is an individual choice for women aged 40‐49 years, while prior to 2009, all women aged 40 years and older were recommended to have mammography every 1‐2 years.^13^ The public response to these guideline changes may affect their mammography screening use.^14^, ^15^, ^16^ Further, different professional societies have different guidelines, which could affect the rates of mammography use.^17^ Thus, continued examination of mammography use over time and its associated factors is necessary. Understanding the complementary evidence from multiple national data sources can better inform policies and intervention strategies for breast cancer screening.^11^, ^12^, ^18^

In the current study, we seek to identify factors associated with mammography use in the entirety of the US population, as well as between black and white subgroups. We also attempt to identify any inconsistency in these factors across subgroups based on NHIS and BRFSS from the same calendar year. To our knowledge, this is the first side‐by‐side comparison of the association between mammography use and risk factors using NHIS and BRFSS. In addition, our selection of risk factors was based on a data‐driven random forest (RF) approach in conjunction with domain knowledge. To facilitate result interpretation, we presented the predictive margins and effect sizes of the associations.^19^, ^20^

## 2 METHODS

### 2.1 Data

We used publicly available 2016 datasets from NHIS and BRFSS, both of which are on‐going, annual, cross‐sectional, nationally representative surveys. NHIS is an in‐person household survey with a multistage sampling design that surveys approximately 87 500 persons in 35 000 households representative of the civilian noninstitutionalized population in the 50 states and the District of Columbia. The response rate was 67.9%, 98.9%, and 80.9% for household, family, and sample adult components, respectively.^21^ BRFSS is a random‐digit‐dialed telephone survey that collects state data from US adults (aged 18 years and older) residents on their health‐related risk behaviors, chronic health conditions, and use of preventive services. Each year more than 400 000 adults are interviewed. The combined landline and cell phone response rates varied by state with a median of 47.1% and a range of 30.7% to 65.0%.^22^ This study used data and materials produced by federal agencies that are in the public domain and did not require Institutional Review Board (IRB) approval.

We extracted self‐reported mammography use in the past year among women from NHIS and BRFSS, which included 12 639 and 169 116 women aged 40‐74 years without a history of breast cancer, respectively. The age range of 40‐74 years was chosen according to the latest USPSTF recommendation in 2016, which is the year both surveys were conducted.^17^ Women aged 75 years and older were excluded, as the USPSTF states a lack of sufficient evidence to assess the balance of benefits and harms of mammography screening for this age group. For women aged 40‐49 years, USPSTF states that the decision to start screening should be individual. We suspect that a substantial percent of women in this age group undergo mammography as they may value the potential benefit of screening more than the potential harm. Thus, we also included this age group in the analysis.

### 2.2 Outcome variable: Past year mammography use

The Yes vs No status of mammography use in the past year was defined based on the participant's response to the question “Have you had a mammogram during the past 12 months?” in NHIS. In BRFSS, it was based on combined responses to the questions “Have you ever had a mammogram?” and “How long since last mammogram?”, where the timing was grouped into: <1 year, 1‐2 years, 2‐3 years, 3‐5 years, and ≥ 5 years. To be consistent with NHIS, we considered participants who answered “Yes” to the first question “Have you ever had a mammogram” and had a mammogram < 1 year as having past year mammography use. In both surveys, those whose answers were “Refused,” “Not ascertained,” or “Don't know” were considered as missing. The choice of using mammography use in the past year, instead of in the past 2 years, is largely constrained by the available matching survey data between NHIS and BRFSS for the calendar year 2016. The most recent data on mammography use in the past 2 years in NHIS are available in the Cancer‐specific module in 2015.^23^ However, in the 2015 BRFSS, the breast and cervical cancer screening modules were only available for a handful of the states and thus are not nationally representative.^24^ To ensure a fair side‐by‐side comparison, we used mammography use in the past year. Past year mammography use has also been included in reports on Cancer Prevention & Early Detection by the American Cancer Society.^25^

### 2.3 Explanatory variables

Eighty‐one variables from each survey (Table S1) that potentially correlated with mammography use were assessed using the criteria of “exactly the same,” “somewhat similar,” and “dissimilar” based on the survey questions and variable definitions, and independently reviewed by three authors (LL, BL, and JJ). Some of the variables were recoded based on the common practice or clinical meaning, and all variables were classified into the following four categories:

#### 2.3.1 Demographic and socioeconomic status

We included 20 and 16 variables from NHIS and BRFSS, respectively (Table S1). We recoded age, education, current employment status, family annual income, number of children, race, marital status, health insurance, and region in BRFSS.

#### 2.3.2 Behavior

There were 10 and 12 variables from NHIS and BRFSS, respectively (Table S1). This included survey respondent's behavioral risk factors or daily habits, such as smoking, drinking, daily exercise, and hours of sleep. Drinking and driving, and wearing a seatbelt was culled from BRFSS. We recoded smoking and drinking status.

#### 2.3.3 Health status

There were 32 and 28 variables from NHIS and BRFSS, respectively (Table S1). We categorized BMI as <24.9, 25‐29.9, 30‐34.9, 35‐39.9, and ≥40 kg/m^2^. We dichotomized the comorbidity variables from both surveys as Yes/No. In addition, we included family member's functional limitation (Yes/No) from NHIS, and activity limitation (Yes/No) from BRFSS.

#### 2.3.4 Healthcare utilization

We included 19 and 24 variables from NHIS and BRFSS, respectively (Table S1). We recoded preventive care screening to include anyone who reported having a pap smear or colon cancer screening. We included a doctor visit during the past 12 months, a dentist visit during the past 2 years, flu shot during past 12 months, blood sugar test during the past 12 months, and an eye exam during the past 2 years. We recoded affordability (Yes/No), if unable to afford medicine or seeing doctors, and delays in getting medical care.

### 2.4 Statistical analysis

**Figure 1:**
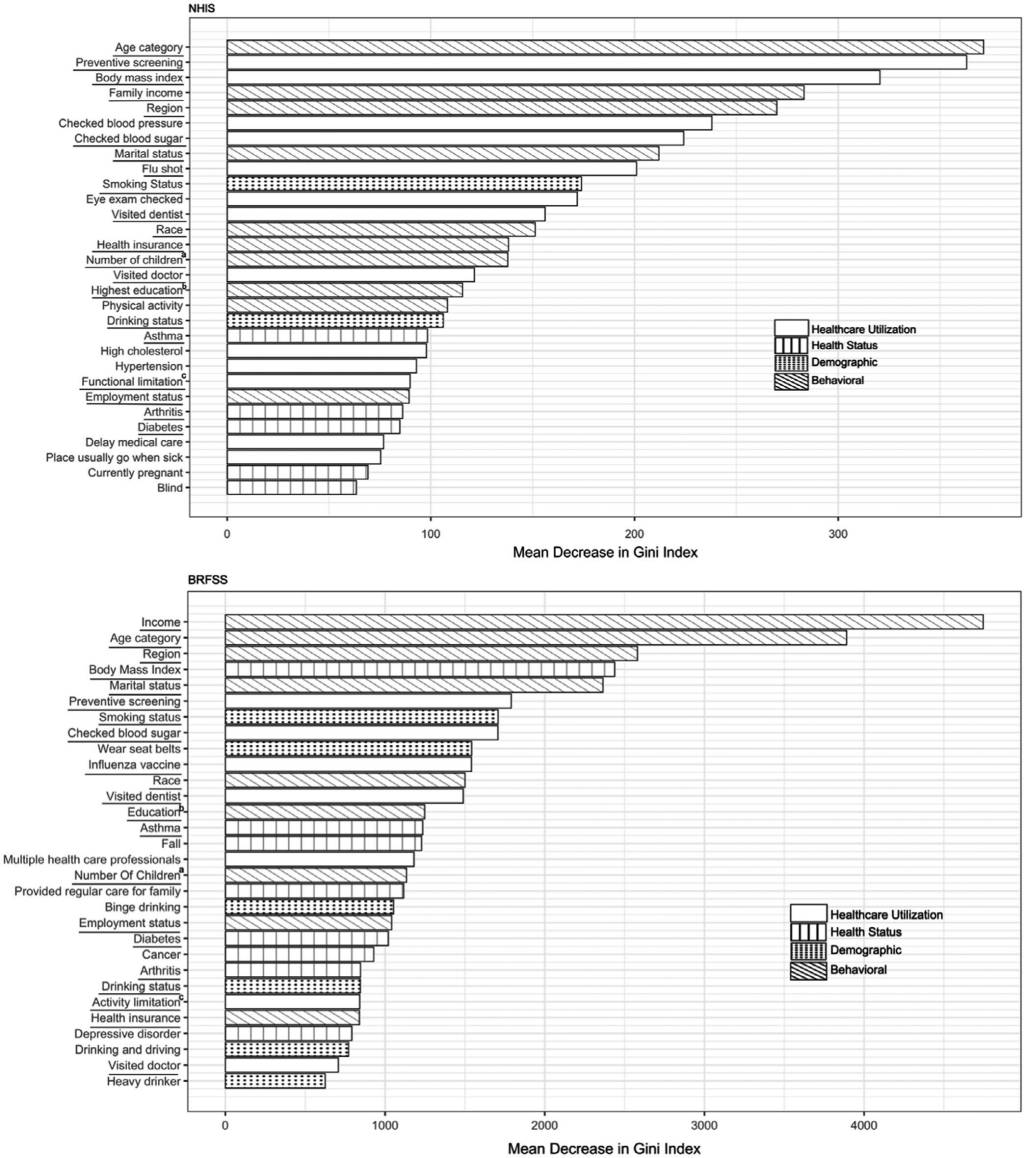
Importance ranking of top 30 factors, identified from random forest algorithm, for predicting past year mammography use from 2016 NHIS and 2016 BRFSS.Note: ,^a^ Number of children in the home; ^b^Highest education in the family; ^c^Functional limitation: Yes (have difficulty walking 1/4 mile, climbing 10 steps, standing for 2 hours, sitting for 2 hours, stooping/bending/kneeling, reaching over head, grasping small objects, lifting/carrying 10lbs, pushing large objects, going out to events, participating in social activities, and relaxing at home without special equipment) in NHIS; Activity limitation: Yes (have serious difficulty walking or climbing stairs, dressing or bathing, doing errands alone because of a physical, mental, or emotional condition) in BRFSS

Variable selection using RF: We used RF with variable importance metrics to identify the 30 most important factors (Figure 1) of the 81 potential factors that predicted mammography use in each survey. The RF is an “ensemble learning” method based on classification and decision trees.^26^ Through aggregating multiple decision trees, it drastically reduces variance compared to fitting a single decision tree and improves prediction accuracy compared to traditional parametric methods. In addition, the RF can calculate variable importance measures, so that variables can be ranked by predictive importance.^26^, ^27^ The variable importance measures are often used to select a subset of variables, while retaining the same prediction accuracy.^27^ We also applied the RF algorithm to subgroups stratified by black and white race, and presented the variable importance plots (Figure[Link], [Link]A,B). The RF analysis was conducted using package “Random Forest” in R (Version 3.5.0, R foundation).

Variable selection for logistic regression models: To further understand the associations between the identified risk factors and mammography use, we conducted a survey logistic regression analysis. We selected a subset of the 30 most important variables that overlapped (n = 21) between the two surveys in the regression analysis for a fair, side‐by‐side comparison. We excluded the following five variables from the regression model: preventive care screening, flu shot, dentist visit, doctor visit, and blood sugar test. These variables were excluded because they can be viewed as the same type of “down‐stream” product as mammography use, similarly impacted by the same set of “up‐stream” factors such as demographic, socioeconomic, health status, and health behavior. Thus, to unpack and quantify the associations between the up‐stream factors and mammography use, we excluded these five specific healthcare utilization factors and used the remaining 16 factors in the final regression model.

Descriptive analyses were performed to compare the distribution of variables between the two surveys. Weighted and unweighted frequencies of risk factors were reported, including the missing response (2% in NHIS and 5% in BRFSS). A multivariable survey logistic regression was then used to examine the association between mammography use and all 16 selected risk factors (age, sex, race, income/family income, education, insurance, marital status, employment, region, family functional limitation/activity limitation, number of children, smoking, drinking status, BMI and diabetes, arthritis, and asthma), while accounting for the complex survey design. Survey logistic regression is an appropriate approach for binary outcomes from survey data with survey elements incorporated, including survey strata, clusters, and weights.^28^, ^29^, ^30^ We applied the logistic regression model to the overall data and to the black and white subpopulations. Predictive margins and their differences were reported to facilitate result interpretation.^28^ Predictive margin, which is a regression estimate on the probability scale rather than the ratio of odds, is interpreted as the average predicted rate of having the outcome. Effect size is the difference of predictive margins, which gives the magnitude of group differences and conveys the scale of difference better than the regression coefficient. The study population in the regression included respondents with non‐missing values, while those with a missing response or covariates were retained in the analysis to preserve the sampling weight and properly calculate the standard errors of the population‐weighted estimates. We also conducted subgroup analysis stratified by age (<50 and ≥ 50 years).

The analysis was performed in STATA (Version 14, College Station, TX). All tests were two‐sided and *P* value < .05 was considered statistically significant.

## 3 RESULTS

### 3.1 Sample characteristics

Comparisons of unweighted samples: A total of 6941 of the 12 639 (54.92%) women aged 40‐74 years in NHIS and 94 274 of the 169 116 (55.75%) in BRFSS self‐reported mammography use in the past year. NHIS respondents were more likely to be younger, with 32.53% below 50 years of age, compared to 19.15% in BRFSS. The NHIS respondents were also more likely to be black (12.85% vs 9.20%), from the Western region of the US (26.03% vs 21.13%), and be current drinkers (61.89% vs 44.58%) than in the BRFSS sample (Table 1).

**Table 1:** Characteristics of study samples from 2016 NHIS and 2016 BRFSS

Variables	NHIS		Variables	BRFSS	
	Unweighted N (%) (N = 12 639)	Weighted N (%) (N = 96 790 834)		Unweighted N (%) (N = 169 116)	Weighted N (%) (N = 67 415 341)
***Demographic***					
**Age** (y)			**Age** (y)		
40 to 44	2108 (16.68)	18 082 266 (18.68)	40 to 44	14 641 ( 8.66)	10 086 383 (14.96)
45 to 49	2003 (15.85)	18 206 508 (18.81)	45 to 49	17 738 (10.49)	9 250 106 (13.72)
50 to 54	1925 (15.23)	16 449 146 (16.99)	50 to 54	22 839 (13.50)	11 571 247 (17.16)
55 to 59	1852 (14.65)	14 423 814 (14.90)	55 to 59	27 224 (16.10)	10 412 367 (15.45)
60 to 64	1792 (14.18)	12 896 619 (13.32)	60 to 64	30 473 (18.02)	10 738 950 (15.93)
65 to 69	1736 (13.74)	9 865 557 (10.19)	65 to 69	31 084 (18.38)	8 611 452 (12.77)
70 to 74	1223 (9.68)	6 866 924 (7.09)	70 to 74	25 117 (14.85)	6 744 836 (10.00)
**Race**			**Race**		
White only	9937 (78.62)	74 276 452 (76.74)	White only	139 848 (82.69)	50 902 431 (75.51)
AIAN only^a^	148 (1.17)	991 284 (1.02)	AIAN only^a^	2904 (1.72)	927 473 (1.38)
Asian only	644 (5.10)	6 789 239 (7.01)	Asian only	2527 (1.49)	3 005 752 (4.46)
Black/AA only^b^	1624 (12.85)	12 931 938 (13.36)	Black or AA only^b^	15 555 (9.20)	8 268 390 (12.26)
Other	246 (1.95)	195 208 (0.20)	Other	5425 (3.21)	2 423 695 (3.60)
Missing	40 (0.32)	1 606 713 (1.66)	Missing	2857 (1.69)	1 887 601 (2.80)
**Marital status**			**Marital status**		
Married	6901 (54.60)	64 175 972(66.30)	Married	98 370 (58.17)	41 965 572 (62.25)
Divorced or separated	3095 (24.49)	17 851 076 (18.44)	Divorced or separated	35 038 (20.72)	13 198 793 (19.58)
Never married	1364 (10.79)	8 434 844 (8.71)	Never married	14 003 (8.28)	5 802 676 (8.61)
Widowed	1256 (9.94)	6 233 462 (6.44)	Widowed	20 877 (12.34)	6 104 931 (9.06)
Missing	23 (0.18)	95 480 (0.10)	Missing	828 (0.49)	343 368 (0.51)
**Highest education** ^c^			**Education** ^c^		
Grade school or high school	3271(25.88)	22 047 027 (22.78)	Grade school or high school	56 397 (33.35)	26 585 624 (39.44)
College or above	9356 (74.02)	74 682 789 (77.16)	College or above	112 382 (66.45)	40 639 050 (60.28)
Missing	12 (0.09)	61 018 (0.06)	Missing	337 (0.20)	190 667 (0.28)
**Employment**			**Employment**		
Unemployed	5584 (44.18)	40 769 518 (42.12)	Unemployed	88 241 (52.18)	32 499 723 (48.21)
Employed	7055 (55.82)	56 021 316 (57.88)	Employed	79 883 (47.24)	34 386 663 (51.01)
			Missing	992 (0.59)	528 955 (0.78)
**Family income**			**Family income**		
$0‐$34,999	3756 (29.72)	22 459 745 (23.20)	$0‐$34,999	52 697 (31.16)	20 949 513 (31.08)
$35,000‐$74,999	3305 (26.15)	23 799 407 (24.59)	$35,000‐$74,999	43 512 (25.73)	16 044 255 (23.80)
$75,000‐$99,999	1377 (10.89)	10 959 997 (11.32)	$75,000 or more	45 729 (27.04)	19 327 777 (28.67)
$100,000 and over	3075 (24.33)	29 771 473 (30.76)	Missing	27 178 (16.07)	11 093 796 (16.46)
Missing	1126 (8.91)	9 800 212 (10.13)			
**Number of children** ^d^			**Number of children** ^d^		
0	8166 (64.61)	56 321 868 (58.19)	0	132 082 (78.10)	46 852 903 (69.50)
1 to 2	3464 (27.41)	31 732 303 (32.78)	1 to 2	29 610 (17.51)	16 270 249 (24.13)
3 or more	1009 (7.98)	8 736 663 (9.03)	3 or more	6327 (3.74)	3 632 749 (5.39)
			Missing	1097 (0.65)	659 440 (0.98)
**Health insurance**			**Health insurance**		
No	873 (6.91)	7 375 370 (7.62)	No	9247 (5.47)	5 556 900 (8.24)
Yes	11 743 (92.91)	89 202 436 (92.16)	Yes	159 524 (94.33)	61 673 538 (91.48)
Missing	23 (0.18)	213 028 (0.22)	Missing	345 (0.20)	184 904 (0.27)
**Region** ^e^			**Region** ^e^		
Northeast	2168 (17.15)	18 456 009 (19.07)	Northeast	35 057 (20.73)	12 368 588 (18.35)
Midwest	2652 (20.98)	20 078 982 (20.74)	Midwest	39 283 (23.23)	13 979 919 (20.74)
South	4529 (35.83)	34 673 893 (35.82)	South	59 038 (34.91)	25 518 360 (37.85)
West	3290 (26.03)	23 581 950 (24.36)	West	35 738 (21.13)	15 548 474 (23.06)
***Behavioral***					
**Smoking status** ^f^			**Smoking status** ^f^		
Current	2056 (16.27)	14 366 914 (14.84)	Current	24 676 (14.59)	10 016 814 (14.86)
Former	2905 (22.98)	20 335 940 (21.01)	Former	44 920 (26.56)	16 229 023 (24.07)
Never	7629 (60.36)	61 752 583 (63.8)	Never	93 458 (55.26)	38 022 329 (56.40)
Missing	49 (0.39)	335 397 (0.35)	Missing	6062 (3.58)	3 147 175 (4.67)
**Drinking status** ^g^			**Drinking status** ^g^		
No	4650 (36.79)	35 495 620 (36.67)	No	84 655 (50.06)	33 339 466 (49.45)
Yes	7822 (61.89)	59 949 764 (61.94)	Yes	77 075 (45.58)	30 262 810 (44.89)
Missing	167 (1.32)	1 345 450 (1.39)	Missing	7386 (4.37)	3 813 065 (5.66)
***Health status***					
**BMI** ^h^			**BMI** ^h^		
Normal or underweight	4240 (33.55)	32 050 760 (33.11)	Normal or underweight	53 361 (31.55)	21 226 043 (31.49)
Overweight	3606 (28.53)	28 437 244 (29.38)	Overweight	47 494 (28.08)	18 623 124 (27.62)
Obese I	2239 (17.72)	16 401 551 (16.95)	Obese Class I	28 422 (16.81)	11 253 644 (16.69)
Obese II	1064 (8.42)	8 396 114 (8.67)	Obese Class II	12 553 (7.42)	5 000 394 (7.42)
Obese III	811 (6.42)	6 029 810 (6.23)	Obese Class III	9543 (5.64)	3 785 042 (5.61)
Missing	679 (5.37)	5 475 355 (5.66)	Missing	17 743 (10.49)	7 527 095 (11.17)
**Functional limitation** ^i^			**Activity limitation** ^i^		
Yes	6393 (50.58)	46 674 854 (48.22)	Yes	37 599 (22.23)	14 912 273 (22.12)
No	6232 (49.31)	50 011 439 (51.67)	No	127 115 (75.17)	50 763 752 (75.30)
Missing	14 (0.11)	104 541 (0.11)	Missing	4402 (2.60)	1 739 316 (2.58)
**Asthma**			**Asthma**		
Current	1454 (11.50)	10 678 795 (11.03)	Current	20 793 (12.30)	8 053 631 (11.95)
Former	529 (4.19)	4 065 944 (4.20)	Former	5688 (3.36)	2 364 467 (3.51)
Never	10 622 (84.04)	81 806 006 (84.52)	Never	141 596 (83.73)	56 655 280 (84.04)
Missing	34 (0.27)	240 089 (0.25)	Missing	1039 (0.61)	341 963 (0.51)
**Arthritis**			**Arthritis**		
No	8037 (63.59)	63 968 857 (66.09)	No	96 228 (56.90)	41 644 793 (61.77)
Yes	4590 (36.32)	32 746 198 (33.83)	Yes	72 135 (42.65)	25 462 884 (37.77)
Missing	12 (0.09)	75 779 (0.08)	Missing	753 (0.45)	307 664 (0.46)
**Diabetes**			**Diabetes**		
No	10 576 (83.68)	81 161 902 (83.85)	No	138 560 (81.93)	54 975 173 (81.55)
Yes	2056 (16.27)	15 542 173 (16.06)	Yes	30 365 (17.96)	12 334 775 (18.30)
Missing	7 (0.06)	86 759 (0.09)	Missing	191 (0.11)	105 393 (0.16)
***Outcome***					
**Mammogram (past 12 mo)**			**Mammogram (past 12 mo)**		
No	5447 (43.10)	42 261 844 (43.66)	No	66 077 (39.07)	26 897 015 (39.90)
Yes	6941 (54.92)	52 563 109 (54.31)	Yes	94 274 (55.75)	35 888 611 (53.24)
Missing	251 (1.99)	1 965 881 (2.03)	Missing	8765 (5.18)	4 629 714 (6.87)

^a^ AIAN = American Indian or Alaskan Native only.

^b^ AA = African American.

^c^ Highest education in the family in NHIS; Education level of individual participant in BRFSS.

^d^ Number of children in the home.

^e^ Region: Northeast (Maine, Vermont, New Hampshire, Massachusetts, Connecticut, Rhode Island, New York, New Jersey, and Pennsylvania); Midwest (Ohio, Illinois, Indiana, Michigan, Wisconsin, Minnesota, Iowa, Missouri, North Dakota, South Dakota, Kansas, and Nebraska); South (Delaware, Maryland, District of Columbia, West Virginia, Virginia, Kentucky, Tennessee, North Carolina, South Carolina, Georgia, Florida, Alabama, Mississippi, Louisiana, Oklahoma, Arkansas, and Texas); West (Washington, Oregon, California, Nevada, New Mexico, Arizona, Idaho, Utah, Colorado, Montana, Wyoming, Alaska, and Hawaii) in both NHIS and BRFSS;

^f^ Smoking status: Current smoker (smoked at least 100 cigarettes in the entire life and is still smoking now); former smoker (smoked at least 100 cigarettes in the entire life but is not smoking now); never (not smoked at least 100 cigarettes in the entire life) in both NHIS and BRFSS;

^g^ Drinking status: Yes (had at least one of any alcoholic beverage during the past 30 d) in NHIS; Yes (had 12 + drinks in lifetime and drinks in past year) in BRFSS.

^h^ BMI = Body mass index, Normal or underweight (BMI ≤ 24.9 kg/m^2^); Overweight (BMI 25‐29.9 kg/m^2^); Obese I (BMI 30‐34.9 kg/m^2^); Obese II (BMI 35‐39.9 kg/m^2^); Obese III ( BMI ≥ 40 kg/m^2^).

^i^ Functional limitation: Yes (have difficulty walking 1/4 mile, climbing 10 steps, standing for 2 hours, sitting for 2 hours, stooping/bending/kneeling, reaching over head, grasping small objects, lifting/carrying 10lbs, pushing large objects, going out to events, participating in social activities, and relaxing at home without special equipment) in NHIS; Activity limitation: Yes (have serious difficulty walking or climbing stairs, dressing or bathing, doing errands alone because of a physical, mental, or emotional condition) in BRFSS.

The unweighted prevalence of past year mammography use was comparable: 54.92% overall, 55.41% in white, and 61.54% in black in NHIS and 55.75% overall, 58.53% in white, and 64.99% in black in BRFSS (Tables 1, 2, 3). The self‐reported mammography use in the black population was consistently higher than that in the white population across all age categories, except for age 60‐64 in NHIS (unweighted 59.53% in white vs unweighted 59.35% in black) (Table S2).

**Table 2:** Associations between risk factors and past year mammography use for all women aged 40‐74 years from 2016 NHIS

Variable	Unweighted prevalence (%)	Weighted prevalence (%)	Predictive margin* (95% CI)	Difference in predictive margin* (95% CI)	*P* value
***Demographic***					
**Age** (y)					
40 to 44	43.88	43.29	45.09 (39.92 to 50.27)		
45 to 49	53.75	53.49	54.34 (49.54 to 59.14)	9.25 (2.71 to 15.78)	.006
50 to 54	58.38	57.85	57.31 (52.98 to 61.65)	12.22 (5.20 to 19.24)	.001
55 to 59	57.21	57.02	55.30 (50.76 to 59.85)	10.21 (2.98 to 17.44)	.006
60 to 64	59.38	57.82	57.50 (52.78 to 62.22)	12.41 (4.90 to 19.92)	.001
65 to 69	64.74	66.22	65.11 (60.66 to 69.57)	20.02 (12.93 to 27.10)	<.001
70 to 74	59.12	60.19	59.93 (54.31 to 65.56)	14.84 (6.48 to 23.19)	.001
**Race**					
White only	55.41	54.50	53.90 (51.89 to 55.91)		
AIAN only^a^	48.87	48.80	61.27 (41.81 to 80.74)	7.38 (−12.37 to 27.12)	.464
Asian only	55.95	51.04	50.19 (41.87 to 58.51)	−3.71 (−12.16 to 4.74)	.389
Black/AA^b^	61.54	61.57	64.07 (58.73 to 69.41)	10.17 (4.26 to 16.09)	.001
Other	48.78	51.34	53.98 (36.42 to 71.55)	0.09 (−17.7 to 17.87)	.992
**Marital status**					
Married	57.43	55.19	54.30 (51.99 to 56.61)		
Divorced or separated	54.76	55.06	57.55 (53.36 to 61.74)	3.25 (−1.67 to 8.17)	.195
Never married	52.22	51.55	53.74 (48.19 to 59.28)	−0.57 (−6.88 to 5.75)	.860
Widowed	55.36	58.17	57.53 (51.52 to 63.53)	3.23 (−3.13 to 9.58)	.319
**Highest education** ^c^					
Grade school or high school	47.73	44.35	48.32 (44.09 to 52.56)		
College or above	58.92	58.28	57.07 (54.99 to 59.15)	8.75 (3.86 to 13.64)	<.001
**Employment**					
Unemployed	54.59	53.41	54.59 (51.6 to 57.59)		
Employed	57.08	56.20	55.38 (53.17 to 57.60)	0.79 (−2.95 to 4.53)	.678
**Family income**					
$0‐$34,999	48.47	47.72	50.75 (46.55 to 54.95)		
$35,000‐$74,999	54.13	50.85	51.08 (48.10 to 54.05)	0.33 (−4.46 to 5.12)	.893
$75,000‐$99,999	62.05	60.02	58.66 (53.43 to 63.90)	7.92 (0.76 to 15.07)	.030
$100,000 and over	64.34	62.02	60.07 (56.08 to 64.07)	9.33 (2.76 to 15.90)	.005
**Number of children** ^d^					
0	58.76	58.75	57.18 (54.68 to 59.67)		
1 to 2	53.42	52.83	54.75 (51.01 to 58.5)	−2.42 (−7.28 to 2.43)	.327
3 or more	43.05	39.42	42.55 (34.88 to 50.21)	−14.63 (−23.13 to −6.13)	.001
**Health insurance**					
No	56.20	33.27	40.22 (33.79 to 46.64)		
Yes	55.96	56.87	56.26 (54.46 to 58.05)	16.04 (9.64 to 22.44)	<.001
**Region** ^e^					
Northeast	62.56	61.63	60.31 (55.55 to 65.07)		
Midwest	56.23	54.89	55.17 (51.43 to 58.91)	−5.13 (−11.03 to 0.76)	.088
South	55.18	54.12	54.60 (51.46 to 57.74)	−5.71 (−11.39 to −0.02)	.049
West	52.69	51.63	51.76 (48.60 to 54.92)	−8.54 (−14.43 to −2.65)	.005
***Behavioral***					
**Smoking status** ^f^					
Current	40.01	40.52	42.74 (38.31 to 47.17)		
Former	59.27	58.97	56.88 (53.11 to 60.64)	14.13 (8.40 to 19.86)	<.001
Never	59.08	57.11	57.29 (54.92 to 59.66)	14.55 (9.35 to 19.75)	<.001
**Drinking status** ^g^					
No	52.22	51.45	53.55 (50.53 to 56.57)		
Yes	58.18	57.11	55.94 (53.71 to 58.18)	2.39 (−1.39 to 6.17)	.215
***Health status***					
**BMI** ^h^					
Normal or underweight	56.30	56.67	55.75 (52.81 to 58.70)		
Overweight	55.81	54.29	53.32 (50.11 to 56.54)	−2.43 (−6.71 to 1.85)	.266
Obese I	57.68	53.87	55.28 (51.14 to 59.42)	−0.47 (−5.69 to 4.74)	.859
Obese II	56.80	58.14	59.98 (53.49 to 66.46)	4.22 (−2.97 to 11.42)	.249
Obese III	49.73	48.90	52.11 (45.37 to 58.84)	−3.65 (−11.17 to 3.88)	.342
**Functional limitation** ^i^					
No	56.58	56.16	56.45 (53.91 to 59.00)		
Yes	55.43	53.83	53.55 (50.65 to 56.45)	−2.90 (−7.01 to 1.20)	.165
**Asthma**					
Current	55.77	56.72	56.91 (51.94 to 61.87)		
Former	57.46	55.26	53.9 (43.91 to 63.88)	−3.01 (−14.02 to 7.99)	.591
Never	55.96	54.80	54.87 (52.84 to 56.9)	−2.03 (−7.64 to 3.57)	.476
**Arthritis**					
No	54.00	53.72	53.75 (51.47 to 56.03)		
Yes	59.47	57.67	57.65 (54.25 to 61.05)	3.90 (−0.41 to 8.22)	.076
**Diabetes**					
No	57.95	55.74	55.59 (53.70 to 57.48)		
Yes	29.74	51.19	52.12 (47.06 to 57.17)	−3.48 (−8.85 to 1.90)	.204

^a^ AIAN = American Indian or Alaskan Native only.

^b^ AA = African American.

^c^ Highest education in the family in NHIS.

^d^ Number of children in the home.

^e^ Region: Northeast (Maine, Vermont, New Hampshire, Massachusetts, Connecticut, Rhode Island, New York, New Jersey, and Pennsylvania); Midwest (Ohio, Illinois, Indiana, Michigan, Wisconsin, Minnesota, Iowa, Missouri, North Dakota, South Dakota, Kansas, and Nebraska); South (Delaware, Maryland, District of Columbia, West Virginia, Virginia, Kentucky, Tennessee, North Carolina, South Carolina, Georgia, Florida, Alabama, Mississippi, Louisiana, Oklahoma, Arkansas, and Texas); West (Washington, Oregon, California, Nevada, New Mexico, Arizona, Idaho, Utah, Colorado, Montana, Wyoming, Alaska, and Hawaii) in NHIS.

^f^ Smoking status: Current smoker (smoked at least 100 cigarettes in the entire life and is still smoking now); former smoker (smoked at least 100 cigarettes in the entire life but is not smoking now); never (not smoked at least 100 cigarettes in the entire life) in both NHIS and BRFSS.

^g^ Drinking status: Yes (had at least one of any alcoholic beverage during the past 30 d) in NHIS.

^h^ BMI = Body mass index, Normal or underweight (BMI ≤ 24.9 kg/m^2^); Overweight (BMI 25‐29.9 kg/m^2^); Obese I (BMI 30‐34.9 kg/m^2^); Obese II (BMI 35‐39.9 kg/m^2^); Obese III( BMI ≥ 40 kg/m^2^).

^i^ Functional limitation: Yes (have difficulty walking 1/4 mile, climbing 10 steps, standing for 2 hours, sitting for 2 hours, stooping/bending/kneeling, reaching over head, grasping small objects, lifting/carrying 10lbs, pushing large objects, going out to events, participating in social activities, and relaxing at home without special equipment) in NHIS.

* The predictive margins accounted for survey strata, cluster, and weight.

**Table 3:** Association between risk factors and past year mammography use for all women aged 40‐74 years from 2016 BRFSS

Variable	Unweighted prevalence (%)	Weighted prevalence (%)	Predictive Margin* (95% CI)	Difference in predictive margin* (95% CI)	*P* value
***Demographic***					
**Age** (y)					
40 to 44	43.15	42.16	44.08 (42.15 to 46.01)		
45 to 49	53.51	53.41	54.31 (52.55 to 56.07)	10.23 (7.8 to 12.65)	<.001
50 to 54	58.62	59.11	59.31 (57.84 to 60.78)	15.23 (12.82 to 17.63)	<.001
55 to 59	59.09	59.09	58.93 (57.42 to 60.43)	14.85 (12.33 to 17.36)	<.001
60 to 64	61.77	60.99	60.58 (59.07 to 62.09)	16.50 (13.90 to 19.10)	<.001
65 to 69	63.89	64.76	62.67 (61.10 to 64.24)	18.59 (15.90 to 21.28)	<.001
70 to 74	62.76	63.69	62.23 (60.36 to 64.11)	18.15 (15.24 to 21.06)	<.001
**Race**					
White only	58.54	56.97	56.27 (55.64 to 56.90)		
AIAN only^a^	51.59	53.79	59.07 (54.25 to 63.89)	2.80 (−2.07 to 7.67)	.260
Asian only	57.41	52.49	51.39 (46.17 to 56.61)	−4.88 (−10.14 to 0.39)	.069
Black/AA^b^	64.99	62.11	64.32 (62.49 to 66.15)	8.05 (6.09 to 10.01)	<.001
Others	54.67	49.75	55.69 (52.26 to 59.11)	−0.58 (−4.08 to 2.92)	.745
**Marital status**					
Married	61.02	58.83	57.76 (56.95 to 58.57)		
Divorced or separated	54.31	52.98	55.92 (54.57 to 57.28)	−1.83 (−3.49 to −0.17)	.030
Never married	55.50	54.29	56.82 (54.72 to 58.92)	−0.94 (−3.26 to 1.38)	.429
Widowed	58.79	57.59	55.58 (53.69 to 57.47)	−2.18 (−4.28 to −0.07)	.043
**Education** ^c^					
Grade school or high school	55.39	53.79	56.80 (55.69 to 57.91)		
College or above	60.44	59.08	57.29 (56.55 to 58.02)	0.48 (−0.91 to 1.88)	.496
**Employment**					
Unemployed	59.35	57.56	57.75 (56.77 to 58.74)		
Employed	58.35	56.78	56.56 (55.67 to 57.45)	−1.20 (−2.66 to 0.26)	.108
**Family income**					
$0‐$34,999	52.21	51.22	53.33 (52.11 to 54.56)		
$35,000‐$74,999	60.28	57.33	55.90 (54.77 to 57.04)	2.57 (0.87 to 4.27)	.003
$75,000 or more	64.82	62.92	61.81 (60.68 to 62.94)	8.48 (6.57 to 10.39)	<.001
**Health insurance**					
No	32.00	32.64	38.85 (36.06 to 41.63)		
Yes	60.28	59.07	58.52 (57.91 to 59.13)	19.68 (16.79 to 22.56)	<.001
**Number of children** ^d^					
0	60.96	59.86	57.89 (57.13 to 58.66)		
1 to 2	52.79	52.18	56.13 (54.76 to 57.50)	−1.76 (−3.45 to −0.07)	.041
3 or more	45.22	44.97	51.77 (48.86 to 54.69)	−6.12 (−9.22 to −3.01)	<.001
**Region** ^e^					
Northeast	62.05	60.45	59.08 (57.80 to 60.35)		
Midwest	59.16	57.84	57.75 (56.75 to 58.76)	−1.32 (−2.93 to 0.28)	.106
South	59.92	57.44	58.01 (56.99 to 59.03)	−1.07 (−2.70 to 0.56)	.200
West	53.75	53.41	53.50 (52.11 to 54.89)	−5.58 (−7.48 to −3.68)	<.001
***Behavioral***					
**Smoking status** ^f^					
Current	44.92	44.57	47.34 (45.83 to 48.85)		
Former	60.24	58.59	56.55 (55.42 to 57.68)	9.20 (7.34 to 11.07)	<.001
Never	62.00	59.97	60.07 (59.29 to 60.85)	12.73 (10.99 to 14.46)	<.001
**Drinking status** ^g^					
No	56.67	55.54	56.48 (55.62 to 57.34)		
Yes	61.05	58.70	57.73 (56.85 to 58.61)	1.25 (−0.04 to 2.53)	.058
***Health status***					
**BMI** ^h^					
Normal or underweight	58.05	56.57	56.81 (55.78 to 57.83)		
Overweight	60.57	58.73	58.08 (57.04 to 59.13)	1.28 (−0.19 to 2.74)	.087
Obese I	59.46	57.08	57.17 (55.79 to 58.55)	0.36 (−1.39 to 2.11)	.685
Obese II	57.96	56.89	56.85 (54.93 to 58.77)	0.04 (−2.17 to 2.26)	.969
Obese III	54.24	53.08	54.24 (51.80 to 56.69)	−2.56 (−5.28 to 0.16)	.065
**Activity limitation** ^i^					
No	58.71	56.97	58.10 (57.41 to 58.80)		
Yes	52.12	54.01	53.46 (51.98 to 54.93)	−4.65 (−6.37 to −2.92)	<.001
**Asthma**					
Current	57.95	56.64	57.72 (56.03 to 59.41)		
Former	57.91	56.79	56.68 (53.55 to 59.81)	−1.04 (−4.57 to 2.50)	.566
Never	59.03	57.29	57.04 (56.40 to 57.68)	−0.68 (−2.49 to 1.13)	.461
**Arthritis**					
No	57.69	55.54	55.61 (54.83 to 56.39)		
Yes	60.44	59.62	59.54 (58.53 to 60.56)	3.93 (2.59 to 5.28)	<.001
**Diabetes**					
No	58.72	56.90	56.81 (56.16 to 57.46)		
Yes	59.32	58.20	58.51 (56.94 to 60.07)	1.70 (−0.03 to 3.42)	.054

^a^ AIAN = American Indian or Alaskan Native only.

^b^ AA = African American.

^c^ Education level of individual participant in BRFSS.

^d^ Number of children in the home.

^e^ Region: Northeast (Maine, Vermont, New Hampshire, Massachusetts, Connecticut, Rhode Island, New York, New Jersey, and Pennsylvania); Midwest (Ohio, Illinois, Indiana, Michigan, Wisconsin, Minnesota, Iowa, Missouri, North Dakota, South Dakota, Kansas, and Nebraska); South (Delaware, Maryland, District of Columbia, West Virginia, Virginia, Kentucky, Tennessee, North Carolina, South Carolina, Georgia, Florida, Alabama, Mississippi, Louisiana, Oklahoma, Arkansas, and Texas); West (Washington, Oregon, California, Nevada, New Mexico, Arizona, Idaho, Utah, Colorado, Montana, Wyoming, Alaska, and Hawaii) in BRFSS.

^f^ Smoking status: Current smoker (smoked at least 100 cigarettes in the entire life and is still smoking now); former smoker (smoked at least 100 cigarettes in the entire life but is not smoking now); never (not smoked at least 100 cigarettes in the entire life) in BRFSS.

^g^ Drinking status: Yes (had 12 + drinks in lifetime and drinks in past year) in BRFSS.

^h^ BMI = Body mass index, Normal or underweight (BMI ≤ 24.9 kg/m^2^); Overweight (BMI 25‐29.9 kg/m^2^); Obese I (BMI 30‐34.9 kg/m^2^); Obese II (BMI 35‐39.9 kg/m^2^); Obese III( BMI ≥ 40 kg/m^2^).

^i^ Activity limitation: Yes (have serious difficulty walking or climbing stairs, dressing or bathing, doing errands alone because of a physical, mental, or emotional condition) in BRFSS.

* The predictive margins accounted for survey strata, cluster, and weight.

Comparison of survey weighted samples: After taking survey weight into account, the similarity of characteristics between the NHIS and BRFSS samples improved overall (Table 1) and for the race‐stratified subgroups (Table S2). The weighted prevalence of past year mammography use was comparable: 54.31% overall, 54.50% in white, and 61.57% in black in NHIS and 53.24% overall, 56.97% in white, and 62.11% in black in BRFSS (Tables 1, 2, 3).

#### 3.1.1 Results from multivariable analysis for overall population

The statistically significant factors associated with mammography use found in both surveys included age, region, number of children at home, income, insurance, black race, and smoking status (Tables 2 and 3). Mammography use prevalence in the West census region of the US was 8.54 percentage points (95% CI, 2.65 ‐ 14.43, *P* = .005) and 5.58 percentage points (95% CI, 3.68‐7.48, *P* < .001) lower than in the Northeast (60.31; 95% CI, 55.55‐65.07 in NHIS; 59.08, 95% CI, 57.80‐60.35 in BRFSS), respectively. Black race was also significantly associated with mammography use (64.07, 95% CI, 58.73‐69.41, *P* = .001 in NHIS; 64.34, 95% CI, 62.49‐66.15, *P* < .001 in BRFSS).

The predicted prevalence of mammography use for black women was approximately 10 percentage points higher than white women (Tables 2 and 3). Women with three or more children in the home also had significantly reduced mammography use. Compared to those with no children at home, those with three or more children had 14.63 (95% CI, 6.13 ‐ 23.13, *P* = .001) and 6.12 (95% CI, 3.01‐9.22, *P* < .001) percentage points lower mammography use in NHIS and BRFSS, respectively. Being a current smoker and lacking insurance were significantly associated with reduced mammography use from both surveys.

Those who were divorced/separated or widowed had lower mammography use in BRFSS (*P* = .030, *P* = .043), but the results were not significant in NHIS (*P* = .195, *P* = .319). The same is true for severe obesity. Likewise, drinking status, activity limitation, and arthritis were suggested to be associated with mammography use in BRFSS but not in NHIS. In contrast, family education was indicated as a significant risk factor in NHIS, with women in a family with a member having college degree or higher 8.75 (95% CI, 3.86‐13.64, *P* < .001) percentage points more likely to report mammography use compared to those in families with highest degree attained being high school or lower. This was not found in BRFSS.

#### 3.1.2 Results from subgroup analysis

Among black women in NHIS, the predicted prevalence of mammography use among those overweight and among those with moderate obesity was 20.03 (95% CI, 6.97‐33.09, *P* = .003) and 19.86 (95% CI, 4.62‐35.10, *P* = .011) percentage points higher than their normal weight counterparts (Table 4). Higher BMI was associated with lower mammography use in BRFSS in the white population (Table 5) with 3.56 (95% CI, 0.47‐6.66, *P* = .024) percentage points lower for women in the severe obese category compared to the normal weight category. There was no significant difference in mammography use by BMI among white women in NHIS. Different from the white population, being divorced or separated did not show any significant association with mammography use among the black population (66.18, 95% CI, 57.67‐74.68, *P* = .394 in NHIS; 63.48, 95% CI, 59.90‐67.06, *P* = .297 in BRFSS). Age, West region, and higher family income were not significant risk factors in the black population in NHIS but were significantly associated with mammography use among blacks in the BRFSS sample. Higher family member's education and having three or more children were associated with mammography use among blacks in the NHIS data but not in BRFSS.

**Table 4:** Associations between risk factors and past year mammography use among white and black women aged 40‐74 years from 2016 NHIS

	NHIS‐White			NHIS‐Black		
Variable	Predictive margin* (95% CI)	Difference in predictive margin* (95% CI)	*P* value	Predictive margin* (95% CI)	Difference in predictive margin* (95% CI)	*P* value
***Demographic***						
**Age (y)**						
40 to 44	43.23 (37.35 to 49.11)			53.00 (39.50 to 66.49)		
45 to 49	53.32 (48.11 to 58.54)	10.09 (2.69 to 17.50)	.008	60.89 (48.38 to 73.41)	7.90 (−9.32 to 25.12)	.368
50 to 54	54.19 (49.36 to 59.01)	10.96 (3.41 to 18.50)	.005	68.87 (57.97 to 79.77)	15.87 (−1.07 to 32.82)	.066
55 to 59	52.81 (47.72 to 57.90)	9.58 (1.24 to 17.91)	.024	67.22 (54.64 to 79.79)	14.22 (−4.38 to 32.82)	.134
60 to 64	58.41 (53.04 to 63.78)	15.18 (6.69 to 23.67)	<.001	58.09 (46.85 to 69.32)	5.09 (−13.22 to 23.40)	.585
65 to 69	64.99 (60.46 to 69.52)	21.76 (14.17 to 29.35)	<.002	61.67 (49.49 to 73.86)	8.68 (−8.83 to 26.18)	.331
70 to 74	60.33 (54.12 to 66.53)	17.09 (7.76 to 26.42)	<.003	63.57 (47.25 to 79.89)	10.58 (−9.94 to 31.09)	.312
**Marital status**						
Married	52.37 (49.97 to 54.77)			61.25 (53.55 to 68.96)		
Divorced or separated	57.98 (53.58 to 62.37)	5.60 (0.57 to 10.63)	.029	66.18 (57.67 to 74.68)	4.92 (−6.42 to 16.27)	.394
Never married	52.26 (44.98 to 59.55)	−0.11 (−7.91 to 7.69)	.978	59.73 (50.03 to 69.43)	−1.52 (−13.32 to 10.27)	.800
Widowed	57.79 (51.66 to 63.91)	5.41 (−0.77 to 11.60)	.086	53.46 (39.24 to 67.69)	−7.79 (−24.55 to 8.97)	.362
**Highest education** ^a^						
Grade school or high school	47.71 (43.02 to 52.40)			45.89 (35.21 to 56.57)		
College or above	55.56 (53.28 to 57.84)	7.85 (2.56 to 13.14)	.004	66.18 (60.27 to 72.09)	20.29 (9.45 to 31.13)	<.001
**Employment**						
Unemployed	53.63 (50.35 to 56.91)			61.37 (52.19 to 70.55)		
Employed	53.83 (51.25 to 56.42)	0.20 (−4.03 to 4.43)	.926	61.65 (55.17 to 68.13)	0.29 (−10.40 to 10.97)	.958
**Family income**						
$0‐$34,999	45.89 (41.21 to 50.57)			63.12 (54.26 to 71.97)		
$35,000‐$74,999	50.02 (46.69 to 53.35)	4.13 (−1.36 to 9.62)	.140	60.26 (51.20 to 69.33)	−2.85 (−13.28 to 7.57)	.591
$75,000‐$99,999	57.25 (51.52 to 62.98)	11.36 (3.67 to 19.05)	.004	64.76 (48.42 to 81.11)	1.65 (−17.24 to 20.53)	.864
$100,000 and over	61.16 (56.92 to 65.40)	15.27 (8.20 to 22.34)	<.001	60.10 (50.34 to 69.86)	−3.02 (−16.77 to 10.74)	.667
**Number of children** ^b^						
0	55.92 (53.28 to 58.57)			65.39 (58.46 to 72.32)		
1 to 2	52.84 (48.35 to 57.33)	−3.09 (−8.68 to 2.51)	.279	62.69 (53.68 to 71.70)	−2.70 (−13.50 to 8.10)	.623
3 or more	43.22 (34.59 to 51.86)	−12.7 (−22.16 to −3.24)	.009	32.60 (14.25 to 50.96)	−32.79 (−51.56 to −14.01)	.001
**Health insurance**						
No	39.88 (32.69 to 47.07)			44.42 (31.25 to 57.58)		
Yes	54.87 (52.79 to 56.94)	14.98 (7.63 to 22.34)	<.001	62.96 (57.24 to 68.68)	18.54 (6.05 to 31.03)	.004
**Region** ^c^						
Northeast	60.85 (55.95 to 65.74)			59.00 (47.63 to 70.38)		
Midwest	54.68 (50.60 to 58.76)	−6.17 (−12.44 to 0.10)	.054	62.87 (50.41 to 75.33)	3.87 (−12.49 to 20.23)	.642
South	52.92 (49.34 to 56.51)	−7.92 (−13.97 to −1.88)	.010	63.19 (57.15 to 69.23)	4.19 (−7.02 to 15.40)	.463
West	48.88 (45.48 to 52.29)	−11.96 (−17.92 to −6.01)	<.001	59.71 (48.07 to 71.35)	0.71 (−15.41 to 16.83)	.931
***Behavioral***						
**Smoking status** ^d^						
Current	42.02 (37.19 to 46.85)			48.90 (36.47 to 61.34)		
Former	55.74 (51.90 to 59.59)	13.72 (7.67 to 19.78)	<.001	63.18 (50.99 to 75.36)	14.27 (−3.09 to 31.63)	.107
Never	55.80 (53.17 to 58.44)	13.78 (8.19 to 19.37)	<.001	63.88 (57.57 to 70.19)	14.98 (1.81 to 28.14)	.026
**Drinking status** ^e^						
No	52.04 (48.45 to 55.63)			62.77 (54.69 to 70.85)		
Yes	54.75 (52.25 to 57.24)	2.71 (−1.74 to 7.16)	.233	60.80 (54.06 to 67.54)	−1.97 (−11.44 to 7.49)	.682
***Health status***						
**BMI** ^f^						
Normal or underweight	54.87 (51.87 to 57.87)			50.51 (39.50 to 61.52)		
Overweight	52.19 (48.78 to 55.61)	−2.68 (−7.20 to 1.85)	.245	70.53 (62.35 to 78.72)	20.03 (6.97 to 33.09)	.003
Obese I	54.25 (49.38 to 59.13)	−0.62 (−6.25 to 5.02)	.830	60.89 (52.30 to 69.48)	10.39 (−3.21 to 23.98)	.134
Obese II	56.11 (48.92 to 63.29)	1.24 (−6.54 to 9.01)	.755	70.37 (59.47 to 81.27)	19.86 (4.62 to 35.10)	.011
Obese III	50.57 (43.52 to 57.62)	−4.30 (−12.05 to 3.44)	.276	65.96 (50.64 to 81.27)	15.45 (−2.48 to 33.38)	.091
**Functional limitation** ^g^						
No	55.42 (52.61 to 58.23)			62.65 (55.26 to 70.04)		
Yes	51.95 (48.85 to 55.04)	−3.47 (−7.79 to 0.85)	.115	60.32 (52.16 to 68.48)	−2.33 (−13.00 to 8.33)	.668
**Asthma**						
Current	53.99 (48.65 to 59.34)			57.44 (41.41 to 73.48)		
Former	57.71 (48.74 to 66.69)	3.72 (−6.49 to 13.92)	.475	39.92 (16.67 to 63.17)	−17.53 (−44.76 to 9.70)	.207
Never	53.52 (51.21 to 55.83)	−0.47 (−6.50 to 5.55)	.877	63.14 (57.43 to 68.86)	5.70 (−9.94 to 21.33)	.475
**Arthritis**						
No	53.17 (50.76 to 55.58)			61.08 (54.30 to 67.86)		
Yes	54.90 (51.30 to 58.50)	1.73 (−2.60 to 6.06)	.432	62.41 (54.20 to 70.63)	1.33 (−8.41 to 11.07)	.789
**Diabetes**						
No	53.79 (51.65 to 55.94)			62.16 (56.25 to 68.06)		
Yes	53.51 (47.88 to 59.14)	−0.28 (−6.31 to 5.75)	.927	58.11 (47.75 to 68.46)	−4.05 (−14.43 to 6.33)	.444

^a^ Highest education in the family in NHIS.

^b^ Number of children in the home.

^c^ Region: Northeast (Maine, Vermont, New Hampshire, Massachusetts, Connecticut, Rhode Island, New York, New Jersey, and Pennsylvania); Midwest (Ohio, Illinois, Indiana, Michigan, Wisconsin, Minnesota, Iowa, Missouri, North Dakota, South Dakota, Kansas, and Nebraska); South (Delaware, Maryland, District of Columbia, West Virginia, Virginia, Kentucky, Tennessee, North Carolina, South Carolina, Georgia, Florida, Alabama, Mississippi, Louisiana, Oklahoma, Arkansas, and Texas); West (Washington, Oregon, California, Nevada, New Mexico, Arizona, Idaho, Utah, Colorado, Montana, Wyoming, Alaska, and Hawaii) in NHIS.

^d^ Smoking status: Current smoker (smoked at least 100 cigarettes in the entire life and is still smoking now); former smoker (smoked at least 100 cigarettes in the entire life but is not smoking now); never (not smoked at least 100 cigarettes in the entire life) in both NHIS and BRFSS.

^e^ Drinking status: Yes (had at least one of any alcoholic beverage during the past 30 d) in NHIS;

^f^ BMI = Body mass index, Normal or underweight (BMI ≤ 24.9 kg/m^2^); Overweight (BMI 25‐29.9 kg/m^2^); Obese I (BMI 30‐34.9 kg/m^2^); Obese II (BMI 35‐39.9 kg/m^2^); Obese III (BMI ≥ 40 kg/m^2^).

^g^ Functional limitation: Yes (have difficulty walking 1/4 mile, climbing 10 steps, standing for 2 hours, sitting for 2 hours, stooping/bending/kneeling, reaching over head, grasping small objects, lifting/carrying 10lbs, pushing large objects, going out to events, participating in social activities, and relaxing at home without special equipment) in NHIS.

* The predictive margins accounted for survey strata, cluster, and weight.

**Table 5:** Associations between risk factors and past year mammography use among white and black women aged 40‐74 years from 2016 BRFSS

	BRFSS‐White			BRFSS‐Black		
Variable	Predictive margin*(95% CI)	Difference in predictive margin* (95% CI)	*P* value	Predictive margin* (95% CI)	Difference in predictive margin* (95% CI)	*P* value
***Demographic***						
**Age** (y)						
40 to 44	43.51 (41.40 to 45.63)			48.12 (42.36 to 53.87)		
45 to 49	53.86 (51.93 to 55.79)	10.35 (7.70 to 12.99)	<.001	57.42 (52.33 to 62.52)	9.31 (2.77 to 15.84)	.005
50 to 54	58.25 (56.69 to 59.82)	14.74 (12.16 to 17.32)	<.001	65.26 (60.72 to 69.80)	17.15 (10.59 to 23.7)	<.001
55 to 59	58.01 (56.47 to 59.55)	14.50 (11.81 to 17.18)	<.001	60.39 (55.65 to 65.14)	12.27 (5.29 to 19.26)	.001
60 to 64	58.97 (57.44 to 60.50)	15.46 (12.69 to 18.22)	<.001	68.01 (63.29 to 72.74)	19.9 (12.65 to 27.14)	<.001
65 to 69	61.57 (60.01 to 63.13)	18.05 (15.22 to 20.89)	<.001	64.99 (59.61 to 70.37)	16.87 (9.04 to 24.70)	<.001
70 to 74	60.92 (59.00 to 62.83)	17.40 (14.32 to 20.49)	<.001	72.66 (65.60 to 79.71)	24.54 (15.29 to 33.79)	<.001
**Marital status**						
Married	56.89 (56.05 to 57.73)			61.05 (57.62 to 64.48)		
Divorced or separated	54.47 (53.03 to 55.91)	−2.42 (−4.14 to −0.69)	.006	63.48 (59.90 to 67.06)	2.44 (−2.14 to 7.01)	.297
Never married	54.80 (52.31 to 57.29)	−2.09 (−4.75 to 0.57)	.124	64.34 (60.53 to 68.15)	3.30 (−1.36 to 7.95)	.165
Widowed	55.72 (53.80 to 57.64)	−1.17 (−3.29 to 0.95)	.278	59.47 (53.56 to 65.39)	−1.57 (−8.23 to 5.08)	.643
**Education** ^a^						
Grade school or high school	55.34 (54.14 to 56.53)			62.70 (59.08 to 66.32)		
College or above	56.57 (55.79 to 57.34)	1.23 (−0.24 to 2.70)	.100	61.16 (58.29 to 64.03)	−1.54 (−5.41 to 2.34)	.437
**Employment**						
Employed	55.49 (54.56 to 56.41)			60.79 (57.52 to 64.06)		
Unemployed	56.83 (55.80 to 57.87)	1.35 (−0.14 to 2.84)	.076	62.87 (59.36 to 66.38)	2.08 (−2.35 to 6.50)	.358
**Family income**						
$0‐$34,999	52.39 (51.08 to 53.70)			56.17 (52.47 to 59.87)		
$35,000‐$74,999	55.14 (53.99 to 56.30)	2.76 (0.98 to 4.53)	.002	61.59 (57.72 to 65.47)	5.42 (0.73 to 10.12)	.024
$75,000 or more	60.69 (59.52 to 61.87)	8.30 (6.31 to 10.3)	<.001	67.24 (62.84 to 71.65)	11.07 (5.27 to 16.88)	<.001
**Number of children** ^b^						
0	56.99 (56.17 to 57.80)			62.65 (59.72 to 65.57)		
1 to 2	55.02 (53.53 to 56.50)	−1.97 (−3.79 to −0.15)	.034	60.52 (56.55 to 64.50)	−2.12 (−6.42 to 2.18)	.333
3 or more	50.13 (46.87 to 53.38)	−6.86 (−10.32 to −3.40)	<.001	56.51 (48.24 to 64.77)	−6.14 (−14.72 to 2.44)	.161
**Health insurance**						
No	36.48 (33.44 to 39.51)			42.41 (35.85 to 48.97)		
Yes	57.66 (56.99 to 58.32)	21.18 (18.04 to 24.33)	<.001	63.33 (60.73 to 65.93)	20.92 (14.56 to 27.27)	<.001
**Region** ^c^						
Northeast	58.14 (56.82 to 59.46)			62.94 (58.71 to 67.17)		
Midwest	56.79 (55.74 to 57.83)	−1.35 (−3.01 to 0.31)	.111	62.98 (59.07 to 66.89)	0.04 (−5.34 to 5.41)	.989
South	56.76 (55.64 to 57.88)	−1.38 (−3.10 to 0.34)	.116	65.98 (63.20 to 68.76)	3.04 (−1.52 to 7.60)	.192
West	52.79 (51.38 to 54.20)	−5.35 (−7.26 to −3.44)	<.001	51.80 (44.51 to 59.10)	−11.14 (−19.31 to −2.97)	.008
***Behavioral***						
**Smoking status** ^d^						
Current	45.91 (44.32 to 47.51)			56.09 (50.86 to 61.32)		
Former	55.52 (54.36 to 56.69)	9.61 (7.63 to 11.59)	<.001	61.22 (56.36 to 66.07)	5.13 (−1.19 to 11.45)	.112
Never	59.16 (58.30 to 60.03)	13.25 (11.38 to 15.12)	<.001	63.51 (60.78 to 66.24)	7.42 (2.15 to 12.69)	.006
**Drinking status** ^e^						
No	55.10 (54.16 to 56.04)			62.76 (59.80 to 65.73)		
Yes	57.11 (56.21 to 58.02)	2.01 (0.68 to 3.34)	.003	60.72 (57.35 to 64.10)	−2.04 (−5.82 to 1.74)	.290
***Health status***						
**BMI** ^f^						
Normal or underweight	56.01 (54.95 to 57.07)			59.30 (54.76 to 63.84)		
Overweight	57.08 (55.98 to 58.18)	1.07 (−0.40 to 2.54)	.154	62.83 (59.49 to 66.16)	3.53 (−1.64 to 8.69)	.181
Obese I	56.26 (54.76 to 57.75)	0.25 (−1.60 to 2.09)	.793	62.98 (58.91 to 67.06)	3.68 (−1.92 to 9.28)	.197
Obese II	55.42 (53.31 to 57.52)	−0.59 (−2.99 to 1.81)	.629	63.63 (58.71 to 68.55)	4.33 (−1.97 to 10.63)	.178
Obese III	52.45 (49.61 to 55.28)	−3.56 (−6.66 to −0.47)	.024	63.44 (58.31 to 68.57)	4.14 (−2.27 to 10.56)	.206
**Activity limitation** ^g^						
No	57.02 (56.27 to 57.77)			62.73 (59.99 to 65.47)		
Yes	52.80 (51.27 to 54.34)	−4.21 (−6.02 to −2.41)	<.001	58.02 (53.47 to 62.58)	−4.70 (−9.42 to 0.01)	.051
**Asthma**						
Current	56.63 (54.80 to 58.46)			63.70 (58.82 to 68.59)		
Former	54.84 (51.35 to 58.32)	−1.79 (−5.71 to 2.12)	.369	59.65 (51.32 to 67.98)	−4.06 (−13.41 to 5.30)	.395
Never	56.09 (55.40 to 56.78)	−0.54 (−2.51 to 1.42)	.587	61.54 (58.83 to 64.25)	−2.17 (−7.11 to 2.77)	.390
**Arthritis**						
No	54.78 (53.94 to 55.63)			59.67 (56.66 to 62.68)		
Yes	58.26 (57.21 to 59.31)	3.48 (2.09 to 4.87)	<.001	65.19 (61.65 to 68.73)	5.52 (1.51 to 9.53)	.007
**Diabetes**						
No	55.79 (55.10 to 56.48)			61.93 (59.27 to 64.60)		
Yes	57.56 (55.88 to 59.25)	1.77 (−0.07 to 3.61)	.059	60.82 (56.57 to 65.07)	−1.11 (−5.23 to 3.01)	.598

^a^ Education level of individual participant in BRFSS.

^b^ Number of children in the home.

^c^ Region: Northeast (Maine, Vermont, New Hampshire, Massachusetts, Connecticut, Rhode Island, New York, New Jersey, and Pennsylvania); Midwest (Ohio, Illinois, Indiana, Michigan, Wisconsin, Minnesota, Iowa, Missouri, North Dakota, South Dakota, Kansas, and Nebraska); South (Delaware, Maryland, District of Columbia, West Virginia, Virginia, Kentucky, Tennessee, North Carolina, South Carolina, Georgia, Florida, Alabama, Mississippi, Louisiana, Oklahoma, Arkansas, and Texas); West (Washington, Oregon, California, Nevada, New Mexico, Arizona, Idaho, Utah, Colorado, Montana, Wyoming, Alaska, and Hawaii) in BRFSS.

^d^ Smoking status: Current smoker (smoked at least 100 cigarettes in the entire life and is still smoking now); former smoker (smoked at least 100 cigarettes in the entire life but is not smoking now); never (not smoked at least 100 cigarettes in the entire life) in BRFSS.

^e^ Drinking status: Yes (had 12 + drinks in lifetime and drinks in past year) in BRFSS;

^f^ BMI = Body mass index, Normal or underweight (BMI ≤ 24.9 kg/m^2^); Overweight (BMI 25‐29.9 kg/m^2^); Obese I (BMI 30‐34.9 kg/m^2^); Obese II (BMI 35‐39.9 kg/m^2^); Obese III ( BMI ≥ 40 kg/m^2^).

^g^ Activity limitation: Yes (have serious difficulty walking or climbing stairs, dressing or bathing, doing errands alone because of a physical, mental, or emotional condition) in BRFSS.

* The predictive margins accounted for survey strata, cluster, and weight.

#### 3.1.3 Results from sensitivity analysis

Subgroup analysis stratified by age (<50 and ≥50 years) yielded consistent results with the main analysis, particularly for women > 50, since they are the majority of the study sample (approximately 70% of study sample in NHIS; 80% of study sample in BRFSS). Results for women aged < 50 years are presented in the Tables [Link], [Link]A,B, [Link], [Link]A,B.

## 4 DISCUSSION

Barriers to mammography use may change over time as targeted public health programs are implemented and screening guidelines are revised. It is therefore critical to periodically assess the data to celebrate public health successes and identify new areas for intervention. Many of the risk factors for mammography use we found are well established in the literature such as age, insurance, income, and smoking status.^4^, ^7^, ^31^, ^32^, ^33^ Our study further validates these factors using two large, nationally representative surveys and a novel machine learning statistical approach. We also identified a change in a well‐established risk factor (race) and identified less known risk factors (parity and region).

### 4.1 Race

Historically, mammography use prevalence for black women has been lower than use among white women.^25^, ^34^ For example, NHIS data showed that the prevalence of mammography use among black women was 5.6%‐7.6% lower than white women during 1987‐1991. The black‐white difference varied between a positive and negative value in alternate years during 1994‐2005, and remained below −0.5% during 2008‐2013. The prevalence of mammography use among black women surpassed that in white women (difference = −4.5%) in 2015.^25^, ^34^ In the current study, we found the prevalence of mammography use was 6% higher among black than white women in both NHIS and BRFSS. Moreover, this change in the historical trend remained statistically significant after controlling for confounding factors in the multivariable regression analysis; black women were more likely to report mammography use in the past year than white women. Changes in mammography use prevalence between white and black women may reflect successful public health campaigns targeting minority women.^25^ For instance, the National Breast and Cervical Cancer Early Detection Program (NBCCEDP), established by the Centers for Disease Control and Prevention (CDC) more than 25 years ago, helps connect low‐income, uninsured, and underserved populations to preventive screening services such as mammography.^35^ CDC also established the African American Women and Mass Media (AAMM) pilot campaign that specifically targets black women and aims to increase mammography use.^36^ Many state and local public health programs targeting disparities in preventive health screening have also been established in response to historically lower screening rates among black women. Our findings suggest that these programs may have been effective in improving mammography use. Targeted education and outreach programs may blunt the effect of conflicting guidelines that may confuse or deter women who are not reached by breast cancer outreach programs. Further research is needed to identify which programs and program components are most effective. Finally, higher mammography use among black women may be influenced by differences in perceived risk or more frequent diagnostic mammography use. Although white women have higher incidence of breast cancer than black women (130.8 per 100 000 vs 126.7/100 000), black women have higher rates of breast cancer mortality (28.4 deaths per 100 000 vs 20.3 deaths per 100 000).^37^

### 4.2 Parity

Different from existing studies, we found that having three or more children in the home is negatively associated with mammography use. This held for the overall population, the white population, and the black population in NHIS data. Although not a common finding, parity has been found to be associated with mammography screening in previous, smaller scale studies. Henry et al, for example, found that having three or more children increased the odds of not receiving mammography in the past 2 years using BRFSS data from Utah.^38^ This finding has been noted internationally among Swedish women as well.^39^ The current study extends these finding to a much larger sample of women using two large surveys. Parity as a risk factor for preventive screening has also been noted for colon cancer, prostate cancer, and cervical cancer according to findings by Stimpson et al, who analyzed 2004‐2006 Medical Expenditure Panel Survey (MEPS) data.^40^

The most logical explanation for the parity findings is constraints on time; as one's role as a caregiver decreases the likelihood of caring for oneself, because women may prioritize care for their children over care for themselves. Childcare may also contribute to the ability to be screened—either through lack of finances to pay for care or social support by family and friends to care for children while the mother is screened. Perceived risk for breast cancer may also play a role. It is possible that women who have more children perceive their risk of breast cancer as low and do not see being screened as important.

Targeting high parity women for mammography use is a public health opportunity. It is likely that women with more than three children have more health‐care encounters than those with fewer or no children. Such encounters, whether in pediatrician offices, urgent care centers, or emergency departments, could be opportunities to educate women and link them to screening opportunities.

### 4.3 Region

Our data analysis revealed that women residing in the West census region of the US had significantly lower mammography use compared to their Northeast counterparts when controlling for other factors. The identified geographic disparities may in part reflect the geographic discrepancies in health‐care resources and their utilization, such as the availability of screening facilities and physician workforce, proportion of urban/rural areas, as well as regional/state differences in the policy/intervention programs for breast cancer screening.^41^, ^42^, ^43^, ^44^, ^45^, ^46^, ^47^, ^48^ The states in the Northeast (ie, Maine, Vermont, New Hampshire, Massachusetts, Connecticut, Rhode Island, New York, New Jersey, and Pennsylvania) tend to have higher density of health‐care resources and higher population densities in general than those in the West (Washington, Oregon, California, Nevada, New Mexico, Arizona, Idaho, Utah, Colorado, Montana, Wyoming, Alaska, and Hawaii). Studies have found that low population density areas tend to have lower mammography capacity and higher travel burden to breast imaging for rural women than urban women.^43^, ^47^

### 4.4 Consistency and incongruence in findings by survey

Many of our findings were consistent across both surveys and it is encouraging to see that validation. Clear, well‐established risk factors for mammography use can provide the public health community with action areas for intervention. For instance, smoking is a risk factor for breast cancer and educators could consider integrating mammography education with smoking cessation programs. Despite the similarity in findings across surveys, there were discrepancies, and this is also important. The findings on obesity are one such example.

Differences in survey mode (face‐to‐face vs phone interview), response rates (81% vs 47%), sample size (12 639 vs 169 116), missing outcome rates (2% vs 5%), and weighting procedures may explain some of the differences observed.^11^, ^49^, ^50^, ^51^, ^52^ Moreover, despite the similarity in weighted sample characteristics between the two surveys, the crude NHIS sample had a higher proportion of black women, women younger than 50 years, and women from the West region than the BRFSS sample. It is important for future researchers to understand these differences when interpreting results and using them to drive public health intervention.^18^

## 5 LIMITATIONS

As in other studies using survey data, our study has some limitations. Survey data were self‐reported and not confirmed by medical record review. Self‐reported data from national surveys may overestimate the screening use compared to data based on health‐care claims.^53^, ^54^, ^55^ Meta‐summaries of studies comparing self‐reported mammogram vs documented screening history showed sensitivity to be between 93% and 95% and specificity to be approximately 62%; the validity and reliability of self‐reported responses also varied across sociodemographic subgroups.^56^, ^57^ In addition, question wording may impact the accuracy of self‐reported mammography. For example, Gonzales et al, (2017) found that mammography use in past year based on a one‐part question, the same as used in the current analysis, tended to be slightly higher than mammography use in the past 2 years obtained when a two‐part question in the NHIS Cancer module was asked; and the inconsistency tended to be higher among women of black race, in poor health, and without a usual source of care.^58^

Mammography can be used for screening or diagnostic purposes and we cannot separate out which reason women received a mammogram using the surveys studied. We excluded women from the sample with a history of breast cancer, but it is possible that some of the mammography reported in the study was diagnostic (ie, the patient had signs or symptoms of breast disease). Our racial disparity discussion is limited to white/black and does not address Hispanic, Asian, or other populations nor does it address immigrant vs native US‐born utilization or English fluency.

## 6 CONCLUSION

Using two large, nationally representative survyes and a novel RF machine learning approach, we identified a change in a previously well‐established risk factor for mammography use as well as less well‐known factors. Our finding that black women were more likely to report mammography use than their white counterparts suggests that public health programs that have sought to increase mammography use among black women may have been effective. This encouraging trend may help narrow the black‐white disparity in breast cancer by detecting cancer early, as later stage at diagnosis accounts for some of the racial disparity in mortality.^59^, ^60^, ^61^ We identified high parity to be negatively associated with mammography use across datasets and found lower prevalence of mammography use in the West region of the US. Our results can be used to inform future federal, state, and local initiatives aimed at improving mammography rates.

## CONFLICT OF INTEREST

We have no conflict of interest to disclose, and there is no financial support for this work that could have influenced our findings.

## AUTHOR CONTRIBUTIONS

Study concept and design, acquisition of data: LL, BL, and JY; Data and statistical analysis: LL, JY, and BL; Drafting of the manuscript: LL, BL, and MB; Result interpretation: LL, BL, and MB; Critical revision of the manuscript for important intellectual content: MB, ET, MM, NB, LRM, LJ, LL, BL, and JY.

## Supporting information

AppendixFig S1A

AppendixFig S1B

AppendixTable S1

AppendixTable S2

AppendixTable S3A

AppendixTable S3B

AppendixTable S4A

AppendixTable S4B

## Data Availability

We used publicly available 2016 datasets from two nationally representative surveys: NHIS and the BFRSS.
