# Supplementary figures and images for: Factors associated with mammography use: A side‐by‐side comparison of results from two national surveys
Source: Cancer Med. 2020 Jul 17;9(17):6430–51. doi: 10.1002/cam4.3128 (PMC7476827; doi:10.1002/cam4.3128)

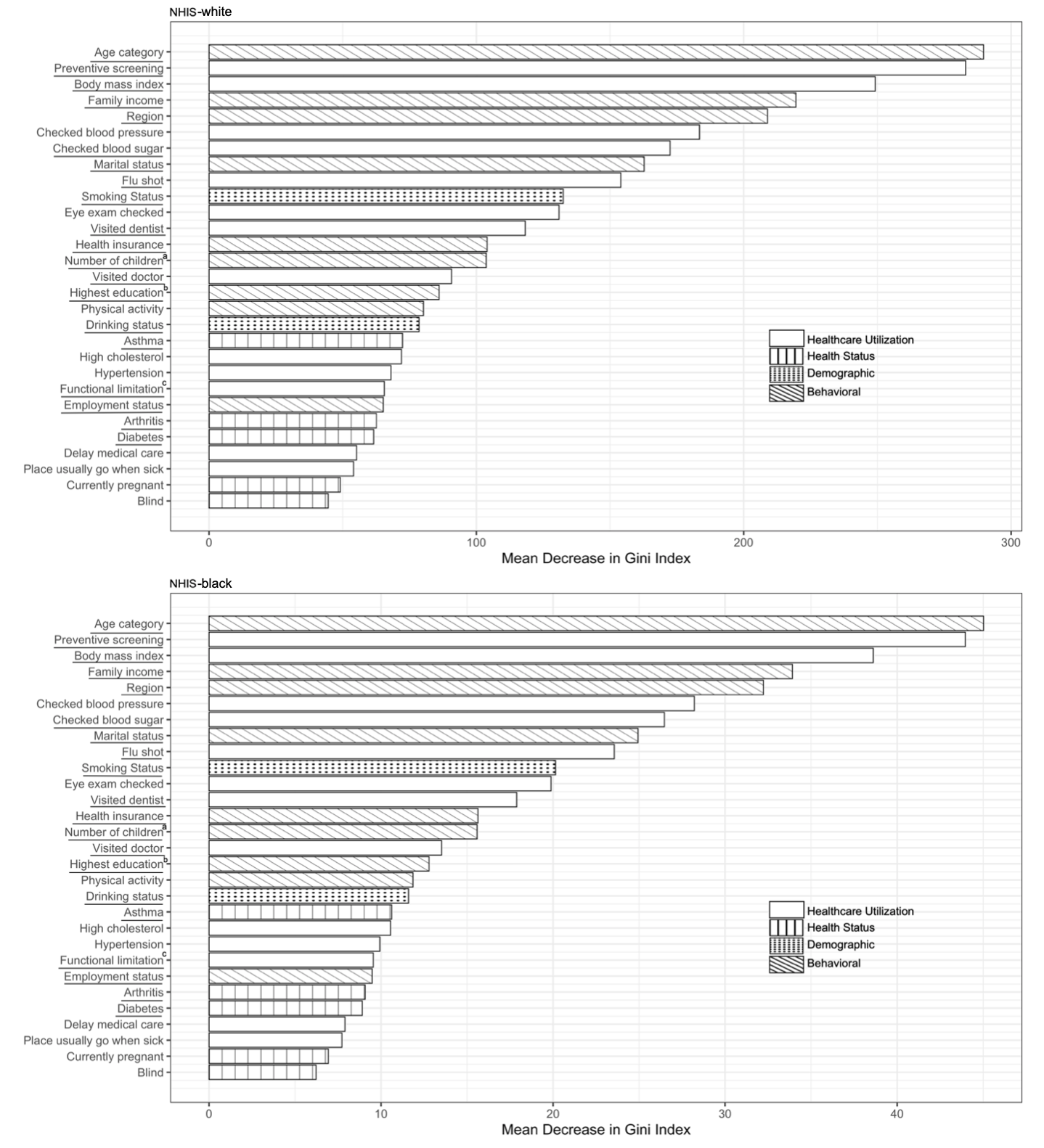

Supplement: Supplementary file 1 — AppendixFig S1A [file CAM4-9-6430-s001.tiff]

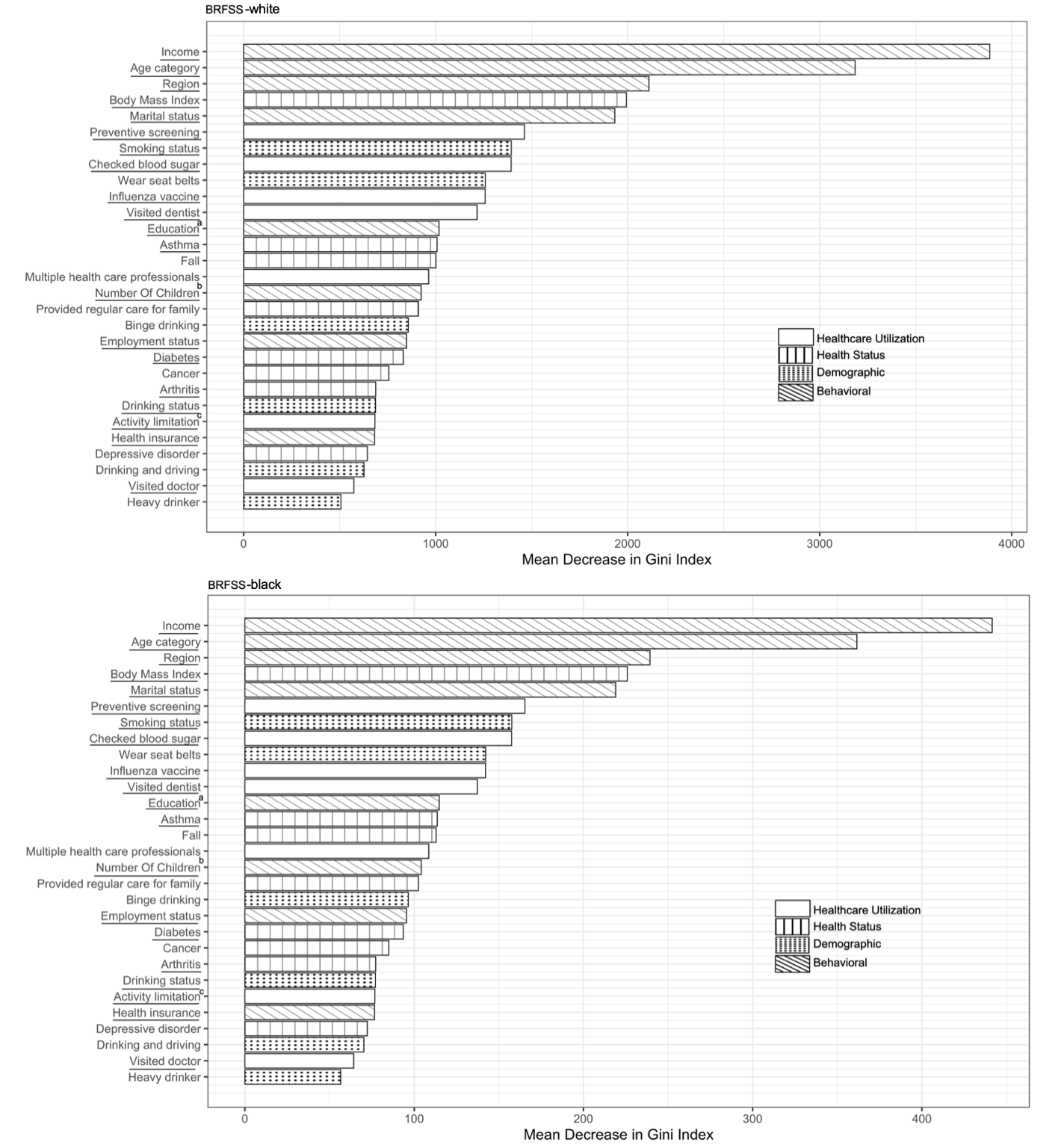

Supplement: Supplementary file 2 — AppendixFig S1B [file CAM4-9-6430-s002.tiff]
