# Supplementary material for: Factors associated with mammography use: A side‐by‐side comparison of results from two national surveys
Source: Cancer Med. 2020 Jul 17;9(17):6430–51. doi: 10.1002/cam4.3128 (PMC7476827; doi:10.1002/cam4.3128)
Supplement: Supplementary file 4 — AppendixTable S2 [file CAM4-9-6430-s004.docx]

**Appendix Table 2.** Prevalence of mammogram use in the past year among white and black women aged 40-74 years from 2016 NHIS and 2016 BRFSS.

|  | | **NHIS-White** | |  | **NHIS-Black** |  | |  | **BRFSS-White** |  | **BRSS-Black** |  |
| --- | --- | --- | --- | --- | --- | --- | --- | --- | --- | --- | --- | --- |
| **Variable** | **Unweighted (%)** | | **Weighted (%)** | | **Unweighted (%)** | | **Weighted (%)** | **Variable** | **Unweighted (%)** | **Weighted (%)** | **Unweighted (%)** | **Weighted (%)** |
| ***Demographic*** |  | |  | |  | |  | ***Demographic*** |  |  |  |  |
| **Age** (years) |  | |  | |  | |  | **Age** (years) |  |  |  |  |
| 40 to 44 | 43.15 | | 41.76 | | 52.91 | | 53.08 | 40 to 44 | 42.84 | 41.56 | 49.21 | 47.51 |
| 45 to 49 | 53.30 | | 53.32 | | 59.42 | | 59.78 | 45 to 49 | 52.88 | 53.30 | 59.68 | 58.03 |
| 50 to 54 | 56.67 | | 55.48 | | 64.65 | | 67.44 | 50 to 54 | 57.74 | 58.30 | 66.87 | 66.52 |
| 55 to 59 | 55.73 | | 55.27 | | 66.00 | | 69.44 | 55 to 59 | 58.46 | 58.83 | 66.56 | 61.31 |
| 60 to 64 | 59.53 | | 59.18 | | 59.35 | | 57.37 | 60 to 64 | 61.31 | 60.27 | 69.54 | 68.98 |
| 65 to 69 | 64.68 | | 66.57 | | 66.30 | | 65.45 | 65 to 69 | 63.59 | 64.40 | 69.11 | 68.93 |
| 70 to 74 | 58.78 | | 60.49 | | 65.14 | | 67.63 | 70 to 74 | 62.47 | 63.40 | 68.22 | 74.03 |
| **Marital status** |  | |  | |  | |  | **Marital status** |  |  |  |  |
| Married | 57.43 | | 55.14 | | 61.83 | | 61.83 | Married | 60.95 | 59.07 | 67.36 | 62.31 |
| Divorced or  Separated | 52.10 | | 53.05 | | 66.74 | | 65.06 | Divorced or Separated | 52.93 | 51.08 | 64.16 | 62.63 |
| Never married | 51.55 | | 47.69 | | 56.47 | | 58.59 | Never married | 53.93 | 51.76 | 61.51 | 60.70 |
| Widowed | 54.87 | | 57.80 | | 57.83 | | 56.55 | Widowed | 58.12 | 57.59 | 66.88 | 63.02 |
| **Highest education^a^** |  | |  | |  | |  | **Education**^a^ |  |  |  |  |
| Grade school or high school | 46.38 | | 41.86 | | 53.71 | | 50.17 | Grade school or high school | 54.48 | 52.68 | 63.18 | 61.08 |
| College or above | 58.35 | | 58.11 | | 66.51 | | 67.31 | College or above | 60.27 | 59.38 | 66.26 | 62.83 |
| **Employment** |  | |  | |  | |  | **Employment** |  |  |  |  |
| Unemployed | 54.25 | | 52.94 | | 59.03 | | 56.18 | Unemployed | 58.10 | 56.81 | 63.88 | 60.27 |
| Employed | 56.28 | | 55.59 | | 63.92 | | 65.92 | Employed | 58.95 | 57.14 | 65.94 | 64.21 |
| **Family income** |  | |  | |  | |  | **Family income** |  |  |  |  |
| $0 - $34,999 | 45.38 | | 43.05 | | 58.58 | | 59.02 | $0 - $34,999 | 50.65 | 49.43 | 61.96 | 59.22 |
| $35,000 - $74,999 | 53.25 | | 50.36 | | 73.68 | | 67.19 | $35,000 - $74,999 | 60.01 | 57.07 | 67.28 | 63.26 |
| $75,000 - $99,999 | 61.55 | | 58.95 | | 62.47 | | 62.17 | $75,000 or more | 64.66 | 63.27 | 70.82 | 68.03 |
| $100,000 and over | 65.12 | | 63.25 | | 64.10 | | 63.28 |  |  |  |  |  |
| **Number of children**^b^ |  | |  | |  | |  | **Number of children**^b^ |  |  |  |  |
| 0 | 58.07 | | 58.21 | | 63.54 | | 65.32 | 0 | 60.54 | 59.63 | 67.40 | 65.13 |
| 1 to 2 | 52.75 | | 51.41 | | 61.32 | | 61.32 | 1 to 2 | 52.15 | 51.61 | 59.58 | 57.77 |
| 3 or more | 42.90 | | 40.49 | | 44.21 | | 39.84 | 3 or more | 44.33 | 44.45 | 53.35 | 49.54 |
| **Health insurance** |  | |  | |  | |  | **Health insurance** |  |  |  |  |
| No | 27.64 | | 31.57 | | 35.59 | | 38.46 | No | 29.52 | 29.85 | 42.54 | 39.36 |
| Yes | 57.33 | | 56.36 | | 64.02 | | 63.81 | Yes | 59.91 | 58.92 | 66.78 | 64.47 |
| **Region**^c^ |  | |  | |  | |  | **Region**^c^ |  |  |  |  |
| Northeast | 63.30 | | 62.90 | | 60.90 | | 61.19 | Northeast | 62.17 | 60.59 | 65.23 | 62.61 |
| Midwest | 57.13 | | 55.69 | | 53.70 | | 55.54 | Midwest | 59.21 | 57.86 | 63.17 | 61.20 |
| South | 53.02 | | 51.87 | | 63.26 | | 62.84 | South | 58.79 | 56.33 | 66.00 | 63.91 |
| West | 51.27 | | 50.67 | | 58.95 | | 61.75 | West | 53.50 | 54.07 | 54.55 | 51.54 |
| ***Behavioral*** |  | |  | |  | |  | ***Behavioral*** |  |  |  |  |
| **Smoking status**^d^ |  | |  | |  | |  | **Smoking status**^d^ |  |  |  |  |
| Current | 39.36 | | 39.17 | | 46.96 | | 50.40 | Current | 43.62 | 43.29 | 56.23 | 53.47 |
| Former | 58.90 | | 59.00 | | 66.07 | | 67.73 | Former | 59.97 | 58.38 | 68.16 | 65.14 |
| Never | 58.54 | | 56.73 | | 64.14 | | 62.72 | Never | 61.88 | 60.33 | 66.18 | 63.56 |
| **Drinking status**^e^ |  | |  | |  | |  | **Drinking status**^e^ |  |  |  |  |
| No | 50.28 | | 49.46 | | 60.90 | | 62.09 | No | 55.61 | 54.29 | 66.02 | 64.05 |
| Yes | 57.94 | | 56.94 | | 62.11 | | 61.15 | Yes | 61.19 | 59.29 | 63.19 | 59.37 |
| ***Health status*** |  | |  | |  | |  | ***Health status*** |  |  |  |  |
| **BMI**^f^ |  | |  | |  | |  | **BMI**^f^ |  |  |  |  |
| Normal or underweight | 56.42 | | 57.60 | | 52.51 | | 47.58 | Normal or underweight | 58.16 | 56.57 | 63.36 | 56.57 |
| Overweight | 55.38 | | 54.05 | | 64.89 | | 69.32 | Overweight | 60.26 | 58.71 | 66.11 | 58.71 |
| Obese I | 56.34 | | 52.82 | | 65.71 | | 58.76 | Obese I | 58.80 | 57.09 | 65.73 | 57.09 |
| Obese II | 54.38 | | 53.88 | | 64.59 | | 67.45 | Obese II | 56.91 | 56.88 | 65.23 | 56.88 |
| Obese III | 48.55 | | 44.88 | | 55.06 | | 66.07 | Obese III | 52.79 | 53.07 | 62.73 | 53.07 |
| **Functional limitation**^g^ |  | |  | |  | |  | **Activity limitation**^g^ |  |  |  |  |
| No | 56.19 | | 56.35 | | 62.02 | | 60.19 | No | 59.21 | 57.66 | 63.04 | 60.78 |
| Yes | 54.63 | | 52.45 | | 61.21 | | 62.60 | Yes | 46.87 | 48.01 | 60.66 | 56.45 |
| **Asthma** |  | |  | |  | |  | **Asthma** |  |  |  |  |
| Current | 55.02 | | 53.74 | | 58.68 | | 62.30 | Current | 57.10 | 55.84 | 66.52 | 63.03 |
| Former | 58.58 | | 58.81 | | 49.09 | | 37.10 | Former | 57.35 | 55.55 | 64.18 | 60.18 |
| Never | 55.30 | | 54.38 | | 62.57 | | 62.87 | Never | 58.78 | 57.18 | 64.76 | 62.02 |
| **Arthritis** |  | |  | |  | |  | **Arthritis** |  |  |  |  |
| No | 53.47 | | 54.01 | | 61.57 | | 60.75 | No | 57.43 | 55.77 | 63.31 | 59.33 |
| Yes | 58.72 | | 55.45 | | 61.50 | | 62.90 | Yes | 60.00 | 58.83 | 67.06 | 66.46 |
| **Diabetes** |  | |  | |  | |  | **Diabetes** |  |  |  |  |
| No | 55.47 | | 55.19 | | 61.36 | | 61.88 | No | 58.65 | 56.91 | 63.88 | 56.91 |
| Yes | 55.01 | | 50.08 | | 62.01 | | 60.57 | Yes | 57.89 | 58.21 | 67.70 | 58.21 |

**Note**: ^a^ Highest education in the family in NHIS; Education level of individual participant in BRFSS;

^b^ Number of Children in the home;

^c^ Region: Northeast (Maine, Vermont, New Hampshire, Massachusetts, Connecticut, Rhode Island, New York, New Jersey, Pennsylvania) ; Midwest(Ohio, Illinois, Indiana, Michigan, Wisconsin, Minnesota, Iowa, Missouri, North Dakota, South Dakota, Kansas, Nebraska); South( Delaware, Maryland, District of Columbia, West Virginia, Virginia, Kentucky, Tennessee, North Carolina, South Carolina, Georgia, Florida, Alabama, Mississippi, Louisiana, Oklahoma, Arkansas, Texas); West(Washington, Oregon, California, Nevada, New Mexico, Arizona, Idaho, Utah, Colorado, Montana, Wyoming, Alaska, Hawaii) in both NHIS and BFRSS;

^d^ Smoking status: Current smoker (smoked at least 100 cigarettes in the entire life and is still smoking now); former smoker (smoked at least 100 cigarettes in the entire life but is not smoking now); never (not smoked at least 100 cigarettes in the entire life) in both NHIS and BRFSS;

^e^ Drinking status: Yes (had at least one of any alcoholic beverage during the past 30 days) in NHIS; Yes (had 12+ drinks in lifetime and drinks in past year) in BRFSS;

^f^ BMI=Body mass index, Normal or underweight (BMI ≤ 24.9 kg/m^2^ ); Overweight(BMI 25–29.9 kg/m^2^); Obese I (BMI 30–34.9 kg/m^2^); Obese II( BMI 35-39.9 kg/m^2^); Obese III( BMI ≥ 40 kg/m2 kg/m^2^);

^g^ Functional limitation: Yes ( have difficulty walking 1/4 mile, climbing 10 steps, standing 2 hours, sitting 2 hours, stooping/bending/kneeling, reaching over head, grasping small objects, lifting/carrying 10lbs, pushing large objects, going out to events, participating in social activities, relaxing at home without special equipment) in NHIS; Activity limitation: Yes (have serious difficulty walking or climbing stair, dressing or bathing, doing errands alone because of a physical, mental, or emotional condition) in BRFSS.
