# Supplementary material for: Factors associated with mammography use: A side‐by‐side comparison of results from two national surveys
Source: Cancer Med. 2020 Jul 17;9(17):6430–51. doi: 10.1002/cam4.3128 (PMC7476827; doi:10.1002/cam4.3128)
Supplement: Supplementary file 5 — AppendixTable S3A [file CAM4-9-6430-s005.docx]

**Appendix Table 3A.** Associations between risk factors and past year mammogram use for all women aged 40-49 years from 2016 NHIS.

| **Variable** | **Unweighted prevalence (%)** | **Weighted prevalence (%)** | **Predictive Margin* (95% CI)** | **Difference in Predictive Margin* (95% CI)** | **P value** |
| --- | --- | --- | --- | --- | --- |
| ***Demographic*** |  |  |  |  |  |
| **Race** |  |  |  |  |  |
| White only | 48.04 | 47.68 | 46.75 (42.15 to 51.35) |  |  |
| AIAN only^a^ | 29.23 | 30.53 | 27.13 (9.70 to 44.57) | -19.62 (-37.29 to -1.94) | 0.030 |
| Asian only | 50.38 | 45.67 | 44.11 (31.50 to 56.71) | -2.64 (-14.92 to 9.63) | 0.672 |
| Black/AA^b^ | 56.05 | 56.29 | 55.57 (44.98 to 66.17) | 8.82 (-2.01 to 19.64) | 0.110 |
| Others | 38.96 | 41.07 | 49.15 (25.02 to 73.28) | 2.40 (-22.37 to 27.16) | 0.849 |
| **Marital status** |  |  |  |  |  |
| Married | 49.48 | 48.14 | 46.58 (41.33 to 51.84) |  |  |
| Divorced or separated | 50.12 | 52.18 | 52.38 (43.78 to 60.99) | 5.80 (-4.23 to 15.83) | 0.257 |
| Never married | 42.19 | 45.62 | 45.65 (35.16 to 56.14) | -0.94 (-13.19 to 11.31) | 0.881 |
| Widowed | 43.28 | 42.01 | 46.22 (23.72 to 68.72) | -0.37 (-23.01 to 22.28) | 0.975 |
| **Highest education**^c^ |  |  |  |  |  |
| Grade school or high school | 39.59 | 35.39 | 37.51 (29.03 to 46.00) |  |  |
| College or above | 50.98 | 51.67 | 50.51 (45.70 to 55.32) | 13.00 (3.74 to 22.26) | 0.006 |
| **Employment** |  |  |  |  |  |
| Unemployed | 40.47 | 42.87 | 46.08 (38.94 to 53.22) |  |  |
| Employed | 51.68 | 50.73 | 48.55 (44.04 to 53.07) | 2.47 (-5.09 to 10.03) | 0.521 |
| **Family Income** |  |  |  |  |  |
| $0 - $34,999 | 41.81 | 43.37 | 46.23 (38.40 to 54.06) |  |  |
| $35,000 - $74,999 | 43.69 | 42.65 | 42.55 (35.70 to 49.40) | -3.68 (-13.44 to 6.08) | 0.460 |
| $75,000 - $99,999 | 55.32 | 55.28 | 52.89 (43.14 to 62.65) | 6.66 (-5.54 to 18.86) | 0.284 |
| $100,000 and over | 55.03 | 52.95 | 50.41 (43.10 to 57.72) | 4.18 (-7.26 to 15.61) | 0.473 |
| **Number of children**^d^ |  |  |  |  |  |
| 0 | 46.58 | 45.72 | 46.35 (40.25 to 52.45) |  |  |
| 1 to 2 | 51.20 | 51.38 | 50.78 (45.79 to 55.76) | 4.42 (-2.65 to 11.50) | 0.220 |
| 3 or more | 44.34 | 43.06 | 43.25 (34.41 to 52.09) | -3.10 (-13.43 to 7.22) | 0.555 |
| **Health Insurance** |  |  |  |  |  |
| No | 30.41 | 32.89 | 36.33 (26.72 to 45.94) |  |  |
| Yes | 50.55 | 50.08 | 48.43 (44.03 to 52.83) | 12.10 (3.04 to 21.16) | 0.009 |
| **Region**^e^ |  |  |  |  |  |
| Northeast | 54.53 | 51.61 | 50.69 (42.42 to 58.97) |  |  |
| Midwest | 51.01 | 52.15 | 51.60 (44.21 to 59.00) | 0.91 (-9.34 to 11.15) | 0.862 |
| South | 48.64 | 48.50 | 46.65 (39.88 to 53.41) | -4.05 (-13.85 to 5.75) | 0.417 |
| West | 43.40 | 42.85 | 42.96 (36.56 to 49.36) | -7.73 (-17.42 to 1.95) | 0.117 |
| ***Behavioral*** |  |  |  |  |  |
| **Smoking Status**^f^ |  |  |  |  |  |
| Current | 37.65 | 41.72 | 41.60 (33.45 to 49.75) |  |  |
| Former | 50.61 | 50.95 | 51.16 (42.96 to 59.37) | 9.56 (-1.65 to 20.77) | 0.094 |
| Never | 50.78 | 49.24 | 47.67 (42.57 to 52.76) | 6.06 (-2.96 to 15.08) | 0.187 |
| **Drinking Status**^g^ |  |  |  |  |  |
| No | 45.82 | 46.63 | 49.16 (43.16 to 55.17) |  |  |
| Yes | 49.82 | 49.21 | 46.61 (41.21 to 52.01) | -2.55 (-9.96 to 4.86) | 0.499 |
| ***Health Status*** |  |  |  |  |  |
| **BMI**^h^ |  |  |  |  |  |
| Normal or underweight | 51.53 | 50.02 | 46.45 (40.68 to 52.22) |  |  |
| Overweight | 45.36 | 45.88 | 44.75 (37.72 to 51.79) | -1.70 (-9.53 to 6.14) | 0.671 |
| Obese I | 50.71 | 51.77 | 53.43 (45.22 to 61.64) | 6.98 (-2.24 to 16.20) | 0.138 |
| Obese II | 51.01 | 54.05 | 53.89 (41.15 to 66.63) | 7.44 (-5.96 to 20.84) | 0.276 |
| Obese III | 41.54 | 42.70 | 41.80 (30.88 to 52.73) | -4.65 (-17.43 to 8.14) | 0.476 |
| **Functional limitation**^i^ |  |  |  |  |  |
| No | 50.02 | 49.09 | 47.75 (42.11 to 53.40) |  |  |
| Yes | 46.12 | 47.19 | 47.30 (40.82 to 53.78) | -0.45 (-8.86 to 7.96) | 0.916 |
| **Asthma** |  |  |  |  |  |
| Current | 46.97 | 46.67 | 45.95 (36.44 to 55.46) |  |  |
| Former | 52.63 | 48.50 | 45.00 (25.65 to 64.36) | -0.94 (-21.92 to 20.03) | 0.930 |
| Never | 48.66 | 48.63 | 47.88 (43.32 to 52.44) | 1.93 (-7.88 to 11.74) | 0.700 |
| **Arthritis** |  |  |  |  |  |
| No | 48.81 | 48.50 | 46.91 (42.37 to 51.45) |  |  |
| Yes | 48.00 | 48.08 | 48.79 (40.02 to 57.56) | 1.88 (-7.69 to 11.45) | 0.700 |
| **Diabetes** |  |  |  |  |  |
| No | 48.96 | 49.07 | 47.99 (43.40 to 52.59) |  |  |
| Yes | 45.48 | 42.26 | 45.00 (34.69 to 55.31) | -2.99 (-13.77 to 7.79) | 0.586 |

**Note**: * The predictive margins accounted for survey strata, cluster and weight;

^a^ AIAN=American Indian or Alaskan Native only;

^b^ AA=African American;

^c^ Highest education in the family in NHIS;

^d^ Number of Children in the home;

^e^ Region: Northeast (Maine, Vermont, New Hampshire, Massachusetts, Connecticut, Rhode Island, New York, New Jersey, Pennsylvania) ; Midwest(Ohio, Illinois, Indiana, Michigan, Wisconsin, Minnesota, Iowa, Missouri, North Dakota, South Dakota, Kansas, Nebraska); South( Delaware, Maryland, District of Columbia, West Virginia, Virginia, Kentucky, Tennessee, North Carolina, South Carolina, Georgia, Florida, Alabama, Mississippi, Louisiana, Oklahoma, Arkansas, Texas); West(Washington, Oregon, California, Nevada, New Mexico, Arizona, Idaho, Utah, Colorado, Montana, Wyoming, Alaska, Hawaii) in NHIS;

^f^ Smoking status: Current smoker (smoked at least 100 cigarettes in the entire life and is still smoking now); former smoker (smoked at least 100 cigarettes in the entire life but is not smoking now); never (not smoked at least 100 cigarettes in the entire life) in both NHIS;

^g^ Drinking status: Yes (had at least one of any alcoholic beverage during the past 30 days) in NHIS;

^h^ BMI=Body mass index, Normal or underweight (BMI ≤ 24.9 kg/m^2^ ); Overweight(BMI 25–29.9 kg/m^2^); Obese I (BMI 30–34.9 kg/m^2^); Obese II( BMI 35-39.9 kg/m^2^); Obese III( BMI ≥ 40 kg/m2 kg/m^2^);

^i^ Functional limitation: Yes ( have difficulty walking 1/4 mile, climbing 10 steps, standing 2 hours, sitting 2 hours, stooping/bending/kneeling, reaching over head, grasping small objects, lifting/carrying 10lbs, pushing large objects, going out to events, participating in social activities, relaxing at home without special equipment) in NHIS.
