# Supplementary material for: Factors associated with mammography use: A side‐by‐side comparison of results from two national surveys
Source: Cancer Med. 2020 Jul 17;9(17):6430–51. doi: 10.1002/cam4.3128 (PMC7476827; doi:10.1002/cam4.3128)
Supplement: Supplementary file 6 — AppendixTable S3B [file CAM4-9-6430-s006.docx]

**Appendix Table 3B.** Associations between risk factors and past year mammogram use for all women aged 40-49 years from 2016 BRFSS

| **Variable** | **Unweighted prevalence (%)** | **Weighted prevalence (%)** | **Predictive margin* (95% CI)** | **Difference in predictive margin* (95% CI)** | **P value** |
| --- | --- | --- | --- | --- | --- |
| ***Demographic*** |  |  |  |  |  |
| **Race** |  |  |  |  |  |
| White only | 45.85 | 46.95 | 49.18 (47.36 to 51.00) |  |  |
| AIAN only^a^ | 38.80 | 39.80 | 52.61 (43.73 to 61.50) | 3.43 (-5.54 to 12.40) | 0.453 |
| Asian only | 41.30 | 42.50 | 43.59 (36.17 to 51.01) | -5.59 (-13.00 to 1.81) | 0.139 |
| Black/AA^b^ | 49.94 | 49.14 | 54.01 (50.17 to 57.85) | 4.83 (0.93 to 8.74) | 0.015 |
| Others | 43.28 | 42.18 | 47.83 (41.59 to 54.07) | -1.35 (-7.64 to 4.94) | 0.674 |
| **Marital status** |  |  |  |  |  |
| Married | 47.25 | 47.26 | 49.68 (47.64 to 51.72) |  |  |
| Divorced or separated | 43.07 | 42.17 | 50.28 (47.27 to 53.30) | 0.61 (-2.84 to 4.05) | 0.731 |
| Never married | 42.64 | 43.54 | 47.05 (43.29 to 50.80) | -2.63 (-6.83 to 1.57) | 0.219 |
| Widowed | 41.93 | 42.13 | 49.34 (41.46 to 57.22) | -0.34 (-8.41 to 7.73) | 0.934 |
| **Highest education**^c^ |  |  |  |  |  |
| Grade school or high school | 48.05 | 48.88 | 47.85 (45.12 to 50.59) |  |  |
| College or above | 39.42 | 39.98 | 50.52 (48.54 to 52.50) | 2.67 (-0.45 to 5.78) | 0.093 |
| **Employment** |  |  |  |  |  |
| Employed | 47.76 | 46.73 | 49.82 (47.91 to 51.74) |  |  |
| Unemployed | 41.12 | 43.11 | 49.23 (46.53 to 51.93) | -0.59 (-3.67 to 2.49) | 0.706 |
| **Family income** |  |  |  |  |  |
| $0 - $34,999 | 39.65 | 39.25 | 47.03 (44.14 to 49.93) |  |  |
| $35,000 - $74,999 | 44.76 | 43.15 | 47.62 (44.85 to 50.39) | 0.58 (-3.24 to 4.41) | 0.765 |
| $75,000 or more | 52.42 | 51.01 | 53.56 (50.94 to 56.17) | 6.52 (2.48 to 10.57) | 0.002 |
| **Health insurance** |  |  |  |  |  |
| No | 26.38 | 25.98 | 30.67 (26.66 to 34.68) |  |  |
| Yes | 47.84 | 46.04 | 51.00 (49.21 to 52.80) | 20.34 (16.16 to 24.51) | <0.001 |
| **Number of children**^d^ |  |  |  |  |  |
| 0 | 46.85 | 45.93 | 50.52 (48.28 to 52.76) |  |  |
| 1 to 2 | 47.05 | 46.15 | 48.19 (46.28 to 50.09) | -2.34 (-5.05 to 0.37) | 0.091 |
| 3 or more | 39.63 | 38.23 | 43.36 (39.99 to 46.72) | -7.17 (-11.10 to -3.23) | <0.001 |
| **Region**^e^ |  |  |  |  |  |
| Northeast | 49.24 | 48.45 | 53.60 (50.66 to 56.54) |  |  |
| Midwest | 46.22 | 47.31 | 51.59 (49.04 to 54.15) | -2.01 (-5.41 to 1.40) | 0.248 |
| South | 47.60 | 46.51 | 51.04 (48.65 to 53.43) | -2.56 (-5.94 to 0.81) | 0.136 |
| West | 38.29 | 38.77 | 42.02 (38.98 to 45.07) | -11.58 (-15.49 to -7.67) | <0.001 |
| ***Behavioral*** |  |  |  |  |  |
| **Smoking status**^f^ |  |  |  |  |  |
| Current | 37.24 | 36.19 | 41.49 (38.39 to 44.59) |  |  |
| Former | 47.24 | 47.35 | 46.75 (43.83 to 49.66) | 5.26 (1.24 to 9.28) | 0.010 |
| Never | 50.69 | 51.19 | 53.02 (50.94 to 55.09) | 11.53 (8.03 to 15.04) | <0.001 |
| **Drinking status**^g^ |  |  |  |  |  |
| No | 45.96 | 44.99 | 49.19 (47.02 to 51.37) |  |  |
| Yes | 49.14 | 48.96 | 49.89 (47.75 to 52.04) | 0.70 (-1.91 to 3.31) | 0.599 |
| ***Health status*** |  |  |  |  |  |
| **BMI**^h^ |  |  |  |  |  |
| Normal or underweight | 47.01 | 46.44 | 49.60 (47.14 to 52.06) |  |  |
| Overweight | 47.51 | 46.93 | 49.22 (46.67 to 51.76) | -0.39 (-3.38 to 2.61) | 0.801 |
| Obese I | 47.35 | 47.05 | 51.10 (48.03 to 54.18) | 1.50 (-2.05 to 5.05) | 0.407 |
| Obese II | 45.98 | 45.09 | 49.14 (45.18 to 53.10) | -0.46 (-4.90 to 3.97) | 0.838 |
| Obese III | 44.34 | 43.95 | 46.82 (42.07 to 51.56) | -2.79 (-8.02 to 2.45) | 0.297 |
| **Activity limitation**^i^ |  |  |  |  |  |
| No | 48.05 | 47.99 | 49.86 (47.85 to 51.87) |  |  |
| Yes | 43.22 | 42.98 | 48.40 (44.90 to 51.91) | -1.46 (-5.56 to 2.64) | 0.486 |
| **Asthma** |  |  |  |  |  |
| Current | 47.55 | 48.01 | 51.88 (48.26 to 55.49) |  |  |
| Former | 44.61 | 43.92 | 46.93 (40.65 to 53.20) | -4.95 (-12.02 to 2.12) | 0.170 |
| Never | 45.56 | 45.09 | 49.33 (47.50 to 51.16) | -2.55 (-6.27 to 1.17) | 0.180 |
| **Arthritis** |  |  |  |  |  |
| No | 45.38 | 44.98 | 47.92 (46.01 to 49.84) |  |  |
| Yes | 47.00 | 46.93 | 52.19 (49.36 to 55.03) | 4.27 (1.11 to 7.43) | 0.008 |
| **Diabetes** |  |  |  |  |  |
| No | 45.82 | 44.99 | 48.82 (47.04 to 50.60) |  |  |
| Yes | 45.12 | 44.93 | 52.97 (49.15 to 56.80) | 4.16 (0.25 to 8.07) | 0.037 |

**Note**: * The predictive margins accounted for survey strata, cluster and weight;

^a^ AIAN=American Indian or Alaskan Native only;

^b^ AA=African American;

^c^ Education level of individual participant in BRFSS;

^d^ Number of Children in the home;

^e^ Region: Northeast (Maine, Vermont, New Hampshire, Massachusetts, Connecticut, Rhode Island, New York, New Jersey, Pennsylvania) ; Midwest(Ohio, Illinois, Indiana, Michigan, Wisconsin, Minnesota, Iowa, Missouri, North Dakota, South Dakota, Kansas, Nebraska); South( Delaware, Maryland, District of Columbia, West Virginia, Virginia, Kentucky, Tennessee, North Carolina, South Carolina, Georgia, Florida, Alabama, Mississippi, Louisiana, Oklahoma, Arkansas, Texas); West(Washington, Oregon, California, Nevada, New Mexico, Arizona, Idaho, Utah, Colorado, Montana, Wyoming, Alaska, Hawaii) in BFRSS;

^f^ Smoking status: Current smoker (smoked at least 100 cigarettes in the entire life and is still smoking now); former smoker (smoked at least 100 cigarettes in the entire life but is not smoking now); never (not smoked at least 100 cigarettes in the entire life) in BRFSS;

^g^ Drinking status: Yes (had 12+ drinks in lifetime and drinks in past year) in BRFSS;

^h^ BMI=Body mass index, Normal or underweight (BMI ≤ 24.9 kg/m^2^ ); Overweight(BMI 25–29.9 kg/m^2^); Obese I (BMI 30–34.9 kg/m^2^); Obese II( BMI 35-39.9 kg/m^2^); Obese III( BMI ≥ 40 kg/m2 kg/m^2^);

^i^ Activity limitation: Yes (have serious difficulty walking or climbing stair, dressing or bathing, doing errands alone because of a physical, mental, or emotional condition) in BRFSS.
