# Supplementary material for: Factors associated with mammography use: A side‐by‐side comparison of results from two national surveys
Source: Cancer Med. 2020 Jul 17;9(17):6430–51. doi: 10.1002/cam4.3128 (PMC7476827; doi:10.1002/cam4.3128)
Supplement: Supplementary file 7 — AppendixTable S4A [file CAM4-9-6430-s007.docx]

**Appendix Table 4A.** Associations between risk factors and past year mammogram use among white and black women aged 40-49 years from 2016 NHIS.

|  | **NHIS-White** |  |  | **NHIS-Black** |  |  | |
| --- | --- | --- | --- | --- | --- | --- | --- |
| **Variable** | **Predictive margin* (95% CI)** | **Difference in predictive margin* (95% CI)** | **P value** | **Predictive margin* (95% CI)** | **Difference in**  **predictive margin***  **(95% CI)** | | **P Value** |
| ***Demographic*** |  |  |  |  |  | |  |
| **Marital status** |  |  |  |  |  | |  |
| Married | 44.50 (39.05 to 49.96) |  |  | 51.85 (37.68 to 66.02) |  | |  |
| Divorced or separated | 54.55 (45.51 to 63.58) | 10.04 (-0.02 to 20.11) | 0.051 | 59.98 (44.04 to 75.91) | 8.13 (-12.32 to 28.57) | | 0.435 |
| Never married | 51.08 (37.58 to 64.57) | 6.57 (-8.16 to 21.31) | 0.381 | 45.08 (30.80 to 59.37) | -6.76 (-28.19 to 14.66) | | 0.535 |
| Widowed | 53.20 (29.80 to 76.60) | 8.70 (-14.66 to 32.05) | 0.465 | 34.42 (-18.58 to 87.41) | -17.43 (-70.34 to 35.48) | | 0.518 |
| **Highest education**^a^ |  |  |  |  |  | |  |
| Grade school or high school | 38.88 (29.27 to 48.49) |  |  | 33.06 (19.69 to 46.43) |  | |  |
| College or above | 49.97 (44.63 to 55.30) | 11.09 (0.66 to 21.53) | 0.037 | 57.44 (45.62 to 69.25) | 24.38 (12.03 to 36.73) | | <0.001 |
| **Employment** |  |  |  |  |  | |  |
| Unemployed | 47.96 (40.06 to 55.86) |  |  | 57.7 (41.81 to 73.59) |  | |  |
| Employed | 47.08 (41.99 to 52.17) | -0.88 (-9.33 to 7.57) | 0.838 | 47.35 (36.07 to 58.63) | -10.34 (-26.23 to 5.55) | | 0.201 |
| **Family income** |  |  |  |  |  | |  |
| $0 - $34,999 | 40.85 (32.20 to 49.50) |  |  | 54.32 (38.89 to 69.74) |  | |  |
| $35,000 - $74,999 | 43.04 (34.90 to 51.17) | 2.19 (-9.00 to 13.38) | 0.701 | 51.15 (33.75 to 68.55) | -3.17 (-19.35 to 13.01) | | 0.701 |
| $75,000 - $99,999 | 53.67 (43.67 to 63.68) | 12.83 (-0.15 to 25.8) | 0.053 | 66.86 (45.79 to 87.92) | 12.54 (-13.16 to 38.24) | | 0.338 |
| $100,000 and over | 53.41 (45.26 to 61.56) | 12.56 (-0.16 to 25.28) | 0.053 | 45.04 (30.47 to 59.6) | -9.28 (-29.78 to 11.22) | | 0.374 |
| **Number of children**^b^ |  |  |  |  |  | |  |
| 0 | 45.41 (38.55 to 52.28) |  |  | 54.88 (40.71 to 69.05) |  | |  |
| 1 to 2 | 51.18 (45.79 to 56.56) | 5.76 (-2.03 to 13.55) | 0.147 | 52.73 (40.93 to 64.52) | -2.15 (-15.78 to 11.47) | | 0.756 |
| 3 or more | 46.82 (37.01 to 56.64) | 1.41 (-10.2 to 13.02) | 0.812 | 24.79 (12.21 to 37.37) | -30.09 (-46.88 to -13.3) | | <0.001 |
| **Health insurance** |  |  |  |  |  | |  |
| No | 35.71 (24.98 to 46.44) |  |  | 39.37 (20.21 to 58.52) |  | |  |
| Yes | 48.37 (43.40 to 53.34) | 12.66 (2.08 to 23.23) | 0.019 | 52.65 (41.45 to 63.85) | 13.28 (-4.06 to 30.62) | | 0.133 |
| **Region**^c^ |  |  |  |  |  | |  |
| Northeast | 53.69 (45.29 to 62.09) |  |  | 45.15 (26.93 to 63.37) |  | |  |
| Midwest | 51.35 (43.29 to 59.4) | -2.34 (-12.97 to 8.29) | 0.666 | 80.09 (63.60 to 96.57) | 34.93 (15.10 to 54.76) | | 0.001 |
| South | 46.37 (38.24 to 54.5) | -7.32 (-18.39 to 3.75) | 0.195 | 47.52 (35.86 to 59.18) | 2.37 (-15.03 to 19.77) | | 0.789 |
| West | 41.07 (34.35 to 47.8) | -12.61 (-22.37 to -2.85) | 0.011 | 35.69 (9.87 to 61.52) | -9.46 (-39.1 to 20.18) | | 0.531 |
| ***Behavioral*** |  |  |  |  |  | |  |
| **Smoking status**^d^ |  |  |  |  |  | |  |
| Current | 41.32 (32.44 to 50.2) |  |  | 45.38 (25.32 to 65.44) |  | |  |
| Former | 53.4 (44.77 to 62.03) | 12.08 (0.23 to 23.94) | 0.046 | 38.43 (15.71 to 61.16) | -6.94 (-34.12 to 20.24) | | 0.616 |
| Never | 46.84 (41.04 to 52.64) | 5.52 (-4.43 to 15.47) | 0.277 | 57.32 (45.93 to 68.70) | 11.94 (-7.57 to 31.44) | | 0.230 |
| **Drinking status**^e^ |  |  |  |  |  | |  |
| No | 48.11 (40.69 to 55.54) |  |  | 57.62 (42.11 to 73.14) |  | |  |
| Yes | 47.06 (41.05 to 53.07) | -1.05 (-10.23 to 8.12) | 0.821 | 48.04 (36.79 to 59.29) | -9.58 (-23.96 to 4.79) | | 0.191 |
| ***Health status*** |  |  |  |  |  | |  |
| **BMI**^f^ |  |  |  |  |  | |  |
| Normal or underweight | 47.65 (41.41 to 53.89) |  |  | 33.01 (18.64 to 47.37) |  | |  |
| Overweight | 44.18 (36.48 to 51.89) | -3.47 (-11.86 to 4.93) | 0.418 | 61.17 (45.09 to 77.25) | 28.16 (8.45 to 47.86) | | 0.005 |
| Obese I | 54.06 (44.53 to 63.58) | 6.41 (-3.89 to 16.70) | 0.222 | 56.05 (40.07 to 72.02) | 23.04 (2.63 to 43.45) | | 0.027 |
| Obese II | 53.83 (39.43 to 68.23) | 6.18 (-9.07 to 21.43) | 0.426 | 67.71 (50.12 to 85.30) | 34.7 (15.18 to 54.23) | | 0.001 |
| Obese III | 34.71 (23.23 to 46.19) | -12.94 (-26.14 to 0.26) | 0.055 | 71.81 (54.03 to 89.59) | 38.8 (19.17 to 58.43) | | <0.001 |
| **Functional limitation**^g^ |  |  |  |  |  | |  |
| No | 46.49 (40.47 to 52.52) |  |  | 60.83 (47.95 to 73.70) |  | |  |
| Yes | 48.48 (41.60 to 55.37) | 1.99 (-6.52 to 10.5) | 0.646 | 41.65 (27.51 to 55.78) | -19.18 (-35.39 to -2.97) | | 0.02 |
| **Asthma** |  |  |  |  |  | |  |
| Current | 42.63 (32.78 to 52.47) |  |  | 53.60 (28.29 to 78.91) |  | |  |
| Former | 51.83 (33.67 to 69.98) | 9.20 (-11.15 to 29.55) | 0.375 | 29.18 (7.21 to 51.15) | -24.42 (-54.68 to 5.85) | | 0.114 |
| Never | 47.85 (42.57 to 53.13) | 5.23 (-5.34 to 15.79) | 0.332 | 52.46 (41.61 to 63.31) | -1.14 (-23.10 to 20.83) | | 0.919 |
| **Arthritis** |  |  |  |  |  | |  |
| No | 47.09 (41.94 to 52.24) |  |  | 51.55 (39.69 to 63.42) |  | |  |
| Yes | 48.14 (39.27 to 57.02) | 1.05 (-8.51 to 10.61) | 0.829 | 51.76 (36.39 to 67.12) | 0.2 (-14.77 to 15.18) | | 0.979 |
| **Diabetes** |  |  |  |  |  | |  |
| No | 46.85 (41.81 to 51.88) |  |  | 54.43 (43.64 to 65.23) |  | |  |
| Yes | 50.76 (38.69 to 62.84) | 3.92 (-8.57 to 16.40) | 0.538 | 37.76 (15.22 to 60.30) | -16.68 (-37.62 to 4.27) | | 0.118 |

**Note**: * The predictive margins accounted for survey strata, cluster and weight;

^a^ Highest education in the family in NHIS;

^b^ Number of Children in the home;

^c^ Region: Northeast (Maine, Vermont, New Hampshire, Massachusetts, Connecticut, Rhode Island, New York, New Jersey, Pennsylvania) ; Midwest(Ohio, Illinois, Indiana, Michigan, Wisconsin, Minnesota, Iowa, Missouri, North Dakota, South Dakota, Kansas, Nebraska); South( Delaware, Maryland, District of Columbia, West Virginia, Virginia, Kentucky, Tennessee, North Carolina, South Carolina, Georgia, Florida, Alabama, Mississippi, Louisiana, Oklahoma, Arkansas, Texas); West(Washington, Oregon, California, Nevada, New Mexico, Arizona, Idaho, Utah, Colorado, Montana, Wyoming, Alaska, Hawaii) in NHIS;

^d^ Smoking status: Current smoker (smoked at least 100 cigarettes in the entire life and is still smoking now); former smoker (smoked at least 100 cigarettes in the entire life but is not smoking now); never (not smoked at least 100 cigarettes in the entire life) in both NHIS;

^e^ Drinking status: Yes (had at least one of any alcoholic beverage during the past 30 days) in NHIS;

^f^ BMI=Body mass index, Normal or underweight (BMI ≤ 24.9 kg/m^2^ ); Overweight(BMI 25–29.9 kg/m^2^); Obese I (BMI 30–34.9 kg/m^2^); Obese II( BMI 35-39.9 kg/m^2^); Obese III( BMI ≥ 40 kg/m2 kg/m^2^);

^g^ Functional limitation: Yes ( have difficulty walking 1/4 mile, climbing 10 steps, standing 2 hours, sitting 2 hours, stooping/bending/kneeling, reaching over head, grasping small objects, lifting/carrying 10lbs, pushing large objects, going out to events, participating in social activities, relaxing at home without special equipment) in NHIS.
