# Supplementary material for: Factors associated with mammography use: A side‐by‐side comparison of results from two national surveys
Source: Cancer Med. 2020 Jul 17;9(17):6430–51. doi: 10.1002/cam4.3128 (PMC7476827; doi:10.1002/cam4.3128)
Supplement: Supplementary file 8 — AppendixTable S4B [file CAM4-9-6430-s008.docx]

**Appendix Table 4B.** Associations between risk factors and past year mammogram use among white and black women aged 40-49 years from 2016 BRFSS.

|  | **BRFSS-White** |  |  | **BRFSS-Black** |  |  |
| --- | --- | --- | --- | --- | --- | --- |
| **Variable** | **Predictive margin* (95% CI)** | **Difference in predictive margin* (95% CI)** | **P value** | **Predictive margin***  **(95% CI)** | **Difference in predictive margin* (95% CI)** | **P value** |
| ***Demographic*** |  |  |  |  |  |  |
| **Marital Status** |  |  |  |  |  |  |
| Married | 49.82 (47.56 to 52.09) |  |  | 44.73 (37.84 to 51.62) |  |  |
| Divorced or separated | 49.25 (45.90 to 52.59) | -0.58 (-4.42 to 3.26) | 0.768 | 55.45 (47.95 to 62.94) | 10.71 (2.62 to 18.81) | 0.010 |
| Never married | 45.48 (40.87 to 50.10) | -4.34 (-9.39 to 0.70) | 0.092 | 50.61 (43.41 to 57.81) | 5.88 (-2.10 to 13.87) | 0.149 |
| Widowed | 47.74 (38.90 to 56.58) | -2.09 (-11.16 to 6.99) | 0.652 | 58.25 (39.14 to 77.35) | 13.52 (-5.80 to 32.84) | 0.170 |
| **Education**^a^ |  |  |  |  |  |  |
| Grade school or high school | 46.94 (43.87 to 50.01) |  |  | 49.23 (41.50 to 56.97) |  |  |
| College or above | 50.45 (48.22 to 52.68) | 3.51 (-0.03 to 7.05) | 0.052 | 48.47 (42.14 to 54.80) | -0.77 (-8.11 to 6.57) | 0.837 |
| **Employment** |  |  |  |  |  |  |
| Unemployed | 49.17 (46.21 to 52.13) |  |  | 47.44 (38.60 to 56.29) |  |  |
| Employed | 49.15 (46.99 to 51.30) | -0.03 (-3.42 to 3.37) | 0.988 | 49.83 (43.77 to 55.88) | 2.39 (-6.74 to 11.51) | 0.608 |
| **Family income** |  |  |  |  |  |  |
| $0 - $34,999 | 47.71 (44.33 to 51.09) |  |  | 42.92 (35.73 to 50.11) |  |  |
| $35,000 - $74,999 | 46.35 (43.29 to 49.41) | -1.36 (-5.78 to 3.06) | 0.546 | 49.64 (41.72 to 57.55) | 6.71 (-1.64 to 15.06) | 0.115 |
| $75,000 or more | 52.87 (50.03 to 55.70) | 5.16 (0.54 to 9.78) | 0.029 | 53.93 (45.32 to 62.55) | 11.01 (0.73 to 21.29) | 0.036 |
| **Number of children**^b^ |  |  |  |  |  |  |
| 0 | 50.08 (47.60 to 52.57) |  |  | 49.43 (42.56 to 56.29) |  |  |
| 1 to 2 | 47.98 (45.86 to 50.11) | -2.10 (-5.08 to 0.89) | 0.169 | 48.08 (41.64 to 54.53) | -1.34 (-8.01 to 5.32) | 0.693 |
| 3 or more | 42.86 (39.02 to 46.70) | -7.22 (-11.7 to -2.75) | 0.002 | 43.70 (34.43 to 52.97) | -5.73 (-15.63 to 4.16) | 0.256 |
| **Health insurance** |  |  |  |  |  |  |
| No | 29.38 (24.58 to 34.18) |  |  | 27.64 (19.62 to 35.67) |  |  |
| Yes | 50.72 (48.71 to 52.73) | 21.34 (16.29 to 26.39) | <0.001 | 50.55 (44.45 to 56.66) | 22.91 (14.78 to 31.04) | <0.001 |
| **Region**^c^ |  |  |  |  |  |  |
| Northeast | 53.20 (50.04 to 56.36) |  |  | 53.63 (45.51 to 61.75) |  |  |
| Midwest | 51.40 (48.68 to 54.12) | -1.8 (-5.4 to 1.79) | 0.325 | 50.40 (41.52 to 59.28) | -3.23 (-13.62 to 7.17) | 0.543 |
| South | 49.31 (46.56 to 52.06) | -3.89 (-7.61 to -0.18) | 0.040 | 54.59 (48.48 to 60.69) | 0.96 (-7.38 to 9.30) | 0.821 |
| West | 43.60 (40.33 to 46.87) | -9.6 (-13.71 to -5.49) | <0.001 | 32.92 (18.78 to 47.06) | -20.71 (-36.02 to -5.4) | 0.008 |
| ***Behavioral*** |  |  |  |  |  |  |
| **Smoking status**^d^ |  |  |  |  |  |  |
| Current | 40.43 (37.08 to 43.77) |  |  | 46.42 (36.01 to 56.82) |  |  |
| Former | 46.66 (43.59 to 49.74) | 6.24 (1.88 to 10.60) | 0.005 | 40.69 (29.38 to 52.00) | -5.73 (-19.33 to 7.87) | 0.409 |
| Never | 52.63 (50.24 to 55.01) | 12.2 (8.27 to 16.13) | <0.001 | 52.80 (46.94 to 58.65) | 6.38 (-3.38 to 16.14) | 0.200 |
| **Drinking status**^e^ |  |  |  |  |  |  |
| No | 48.80 (46.34 to 51.26) |  |  | 49.31 (42.61 to 56.00) |  |  |
| Yes | 49.51 (47.17 to 51.86) | 0.72 (-2.17 to 3.61) | 0.627 | 48.21 (41.50 to 54.91) | -1.1 (-7.45 to 5.25) | 0.734 |
| ***Health status*** |  |  |  |  |  |  |
| **BMI**^f^ |  |  |  |  |  |  |
| Normal or underweight | 49.64 (46.97 to 52.32) |  |  | 45.97 (37.07 to 54.88) |  |  |
| Overweight | 48.01 (45.19 to 50.83) | -1.63 (-4.84 to 1.57) | 0.318 | 51.31 (43.99 to 58.62) | 5.33 (-4.09 to 14.76) | 0.267 |
| Obese I | 51.19 (47.71 to 54.67) | 1.55 (-2.36 to 5.46) | 0.438 | 49.55 (41.08 to 58.03) | 3.58 (-6.67 to 13.84) | 0.494 |
| Obese II | 47.78 (43.19 to 52.37) | -1.87 (-6.91 to 3.18) | 0.469 | 51.57 (42.42 to 60.72) | 5.60 (-5.56 to 16.75) | 0.325 |
| Obese III | 47.88 (42.30 to 53.46) | -1.76 (-7.89 to 4.36) | 0.573 | 45.56 (36.14 to 54.98) | -0.41 (-11.64 to 10.82) | 0.943 |
| **Activity limitation**^g^ |  |  |  |  |  |  |
| No | 49.50 (47.24 to 51.76) |  |  | 48.78 (42.24 to 55.31) |  |  |
| Yes | 47.90 (43.98 to 51.82) | -1.60 (-6.23 to 3.03) | 0.499 | 48.64 (39.46 to 57.82) | -0.14 (-10.12 to 9.84) | 0.979 |
| **Asthma** |  |  |  |  |  |  |
| Current | 49.39 (45.29 to 53.48) |  |  | 58.73 (49.17 to 68.30) |  |  |
| Former | 45.76 (38.79 to 52.74) | -3.62 (-11.53 to 4.29) | 0.370 | 39.95 (24.78 to 55.12) | -18.78 (-35.75 to -1.82) | 0.030 |
| Never | 49.27 (47.22 to 51.31) | -0.12 (-4.38 to 4.15) | 0.957 | 47.70 (41.55 to 53.85) | -11.03 (-20.04 to -2.02) | 0.016 |
| **Arthritis** |  |  |  |  |  |  |
| No | 47.18 (45.05 to 49.31) |  |  | 46.80 (40.5 to 53.11) |  |  |
| Yes | 52.39 (49.21 to 55.57) | 5.21 (1.64 to 8.77) | 0.004 | 52.01 (43.71 to 60.31) | 5.21 (-3.01 to 13.42) | 0.214 |
| **Diabetes** |  |  |  |  |  |  |
| No | 48.36 (46.38 to 50.35) |  |  | 49.28 (43.24 to 55.33) |  |  |
| Yes | 52.85 (48.37 to 57.33) | 4.49 (-0.13 to 9.10) | 0.057 | 46.31 (37.09 to 55.53) | -2.97 (-11.42 to 5.48) | 0.491 |

**Note**: * The predictive margins accounted for survey strata, cluster and weight;

^a^ Education level of individual participant in BRFSS;

^b^ Number of Children in the home;

^c^ Region: Northeast (Maine, Vermont, New Hampshire, Massachusetts, Connecticut, Rhode Island, New York, New Jersey, Pennsylvania) ; Midwest(Ohio, Illinois, Indiana, Michigan, Wisconsin, Minnesota, Iowa, Missouri, North Dakota, South Dakota, Kansas, Nebraska); South( Delaware, Maryland, District of Columbia, West Virginia, Virginia, Kentucky, Tennessee, North Carolina, South Carolina, Georgia, Florida, Alabama, Mississippi, Louisiana, Oklahoma, Arkansas, Texas); West(Washington, Oregon, California, Nevada, New Mexico, Arizona, Idaho, Utah, Colorado, Montana, Wyoming, Alaska, Hawaii) in BFRSS;

^d^ Smoking status: Current smoker (smoked at least 100 cigarettes in the entire life and is still smoking now); former smoker (smoked at least 100 cigarettes in the entire life but is not smoking now); never (not smoked at least 100 cigarettes in the entire life) in BRFSS;

^e^ Drinking status: Yes (had 12+ drinks in lifetime and drinks in past year) in BRFSS;

^f^ BMI=Body mass index, Normal or underweight (BMI ≤ 24.9 kg/m^2^ ); Overweight(BMI 25–29.9 kg/m^2^); Obese I (BMI 30–34.9 kg/m^2^); Obese II( BMI 35-39.9 kg/m^2^); Obese III( BMI ≥ 40 kg/m2 kg/m^2^);

^g^Activity limitation: Yes (have serious difficulty walking or climbing stair, dressing or bathing, doing errands alone because of a physical, mental, or emotional condition) in BRFSS.
